# Using Composite Phenotypes to Reveal Hidden Physiological Heterogeneity in High-Altitude Acclimatization in a Chinese Han Longitudinal Cohort

**DOI:** 10.1007/s43657-020-00005-8

**Published:** 2021-02-22

**Authors:** Yi Li, Yanyun Ma, Kun Wang, Menghan Zhang, Yi Wang, Xiaoyu Liu, Meng Hao, Xianhong Yin, Meng Liang, Hui Zhang, Xiaofeng Wang, Xingdong Chen, Yao Zhang, Wenyuan Duan, Longli Kang, Bin Qiao, Jiucun Wang, Li Jin

**Affiliations:** 1 State Key Laboratory of Genetic Engineering, Collaborative Innovation Center for Genetics and Development, School of Life Sciences and Human Phenome Institute, Fudan University, Shanghai, 200438 China; 2 Ministry of Education Key Laboratory of Contemporary Anthropology, Department of Anthropology and Human Genetics, School of Life Sciences, Fudan University, Shanghai, 200438 China; 3 Institute for Six-Sector Economy, Fudan University, Shanghai, 200433 China; 4 Fudan-Taizhou Institute of Health Sciences, Taizhou, 225300 China; 5 Key Laboratory of High Altitude Environment and Genes Related To Diseases of Tibet Autonomous Region, School of Medicine, Xizang Minzu University, Xianyang, 712082 China; 6 Institute of Cardiovascular Disease, Shandong Provincial Western Hospital, Jinan, Shandong 250022 China; 7 Research Unit of Dissecting the Population Genetics and Developing New Technologies for Treatment and Prevention of Skin Phenotypes and Dermatological Diseases (2019RU058), Chinese Academy of Medical Sciences, Beijing, 100730 China

**Keywords:** Altitude acclimatization, Composite phenotypes, Hypoxia, Complex traits, Phenomics

## Abstract

Altitude acclimatization is a human physiological process of adjusting to the decreased oxygen availability. Since several physiological processes are involved and their correlations are complicated, the analyses of single traits are insufficient in revealing the complex mechanism of high-altitude acclimatization. In this study, we examined these physiological responses as the composite phenotypes that are represented by a linear combination of physiological traits. We developed a strategy that combines both spectral clustering and partial least squares path modeling (PLSPM) to define composite phenotypes based on a cohort study of 883 Chinese Han males. In addition, we captured 14 composite phenotypes from 28 physiological traits of high-altitude acclimatization. Using these composite phenotypes, we applied k-means clustering to reveal hidden population physiological heterogeneity in high-altitude acclimatization. Furthermore, we employed multivariate linear regression to systematically model (Models 1 and 2) oxygen saturation (SpO_2_) changes in high-altitude acclimatization and evaluated model fitness performance. Composite phenotypes based on Model 2 fit better than single trait-based Model 1 in all measurement indices. This new strategy of using composite phenotypes may be potentially employed as a general strategy for complex traits research such as genetic loci discovery and analyses of phenomics.

**Electronic supplementary material:**

The online version of this article (10.1007/s43657-020-00005-8) contains supplementary material, which is available to authorized users.

## Introduction

Altitude acclimatization is a human physiological process of adjusting to decreased oxygen availability (West et al. 2012). It comprises several physiological responses, including ventilation function, cardiac function, oxygen delivery function, hematology, muscle structure and metabolism, and oxygen consumption (Muza et al. 2010; Martin et al. 2010). The most important physiological responses involve the cardiorespiratory and the hematology system (Muza et al. 2010). Oxygen saturation (SpO_2_) reflects the most straightforward physiological changes (Muza et al. 2010; West 2004, 2017; Martin et al. 2014; Peacock and Jones 1997). SpO_2_ rapidly decreased in lowlanders within 3 days of directly ascending to 4300 m, followed by a rise in altitude over weeks (West et al. 2012; Muza et al. 2010; Lundby et al. 2004; Peng et al. 2013). Another well-known physiological change is hemoglobin concentration in the blood (West et al. 2012; Lundby et al. 2004; Peng et al. 2013; Brierley et al. 2012). It is also known that individuals vary in both speed and extent of altitude acclimatization (West et al. 2012; Brown and Grocott 2013; Harper 2010). The variations in responses across individuals provide an opportunity to explore the mechanism of altitude acclimatization (West et al. 2012; Peng et al. 2013; Brown and Grocott 2013).

Since several physiological processes are involved and their correlations are complicated, analyses of single traits are insufficient to capture the complex mechanism of high-altitude acclimatization (West et al. 2012; West 2004; Peng et al. 2013). Therefore, analysis of composite phenotypes, i.e., combinations of physiological phenotypes, could become a promising alternative (Inglese et al. 2017; Ried et al. 2016; Holmes et al. 2008). There are several methods to extract composite phenotypes from multiple traits such as principal component analysis (PCA)-based methods (Ried et al. 2016; Yang et al. 2012; Aschard et al. 2014) and partial least squares (PLS)-based methods (Peng et al. 2013; Li et al. 2013; Zhang et al. 2013). PLS-based methods have better performance than PCA-based methods (Li et al. 2013; Zhang et al. 2013). Partial least squares path modeling (PLSPM) is the PLS-based approach to structural equation modeling (Sanchez 2013; Tenenhaus et al. 2005; Esposito Vinzi et al. 2010), which can also be viewed as a method for analyzing multiple relationships among groups of variables. In the PLSPM framework, there are generally two ways to define composite phenotypes, i.e., latent variables (Peng et al. 2013; Zhang et al. 2013; Sanchez 2013; Tenenhaus et al. 2005; Esposito Vinzi et al. 2010),one is using the prior knowledge, and the other is using data-driven methods such as spectral clustering (Hastie et al. 2009; Luxburg 2007).

**Fig. 1:**
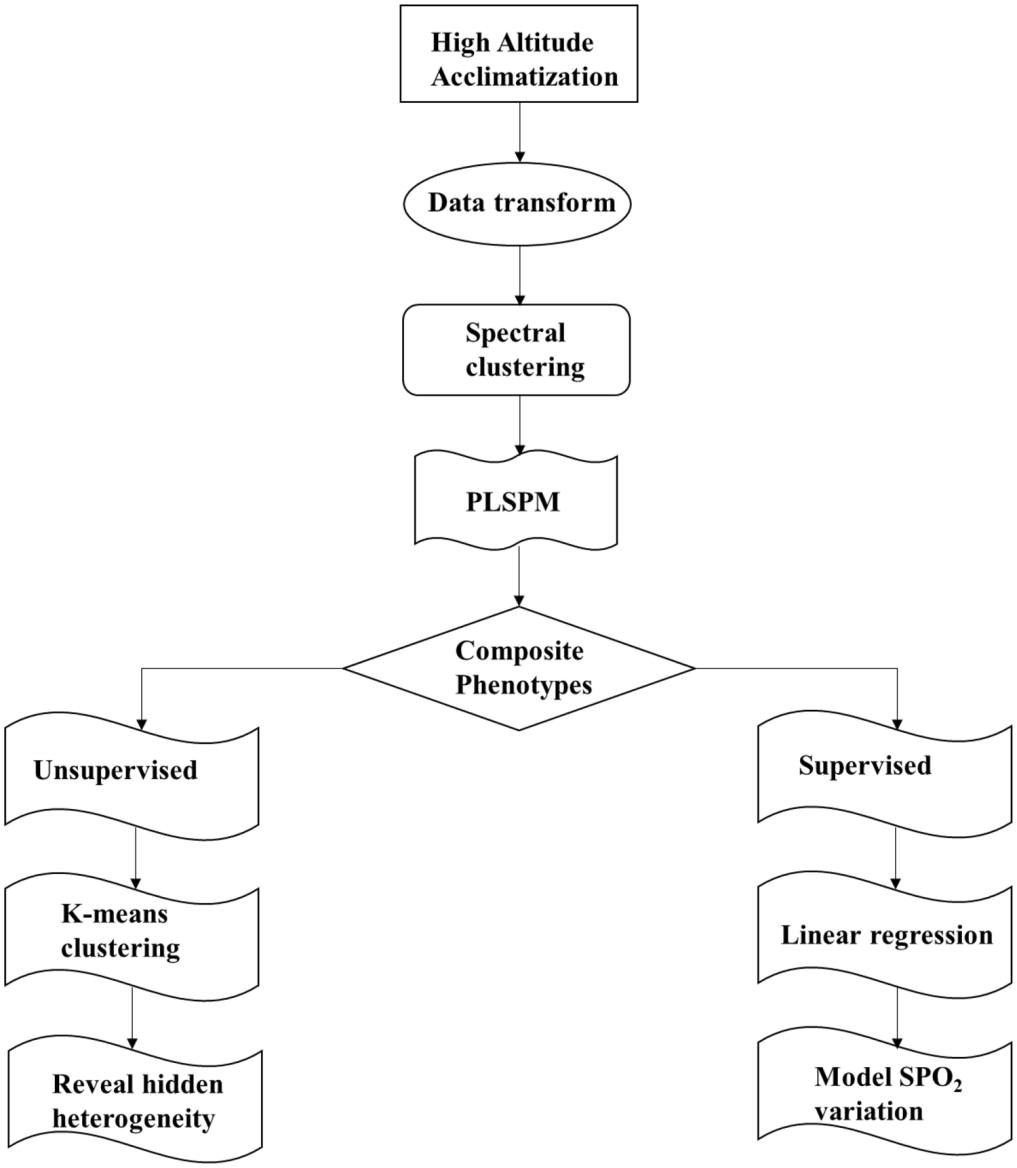
The workflow and design of this study

Here, we conducted a two-phase longitudinal study of high-altitude acclimatization (baseline and chronic phase) in a large sample of 883 Chinese Han young males. A total of 28 physiological phenotypes were collected from these individuals at each phase. Firstly, we extracted composite phenotypes from physiological phenotypes in high-altitude acclimatization by introducing a data-driven strategy constituting spectral clustering (Hastie et al. 2009; Luxburg 2007) and the PLSPM (Sanchez 2013; Tenenhaus et al. 2005) algorithm. Besides, using these composite phenotypes, we revealed hidden population physiological heterogeneity in high-altitude acclimatization using k-means clustering (Luncien et al. 1967). Furthermore, we modeled changes in SpO_2_ during high-altitude acclimatization using multivariate linear regression (Freedman 2009) and further evaluated the advantages of composite phenotypes over single phenotypes. The workflow is summarized in Fig. 1, which is also the design of this study. The term ‘phenotype’ used in this manuscript refers to ‘The Extended Phenotype’ (Dawkins 1978).

## Materials and Methods

### Participants

We conducted a longitudinal cohort measurement design to investigate the responses of 28 physiological traits during high-altitude acclimatization. The subjects were first assembled at a location with an altitude of 50 m (in China) for 10–14 days, and then they arrived at highland of above 4300 m (in China) by train. The study comprised two phases: baseline phase (before going to highland) and chronic phase (living at highland for about 1 month). A structured questionnaire and physiological examination for the subjects were conducted at two phases of high-altitude acclimatization. The subjects with cancer, diabetes, and coronary heart disease were not included in this study. A total of 883 healthy Chinese Han young males with ages from 17 to 36 years were recruited. The research was approved by the Human Ethics Committee of Fudan University, and the written informed consent was obtained from each participant or their guardians who were over 18 years old.

### Physiological Measurements

All the subjects (883 samples, 28 traits) were measured by physicians in Shandong Provincial Western Hospital, who were previously trained to administer both the questionnaire and the physical examination. Systolic blood pressure (SBP) and diastolic blood pressure (DBP) were calculated by mean of two rounds of measurement of a standardized mercury sphygmomanometer. Maximal vital capacity (FVC) was measured by SPIDA5. Heart rate (HR) was determined by measuring radial pulse twice, and SPO_2_ was measured by Nellcor NPB-40. The body temperature (Temperature) was measured by a thermometer. The blood specimens were drawn after overnight fasting for complete blood count measurement by a three-classification haemacytometer analyzer (Model CA-800; CIS, Japan). The blood routine indices included red blood cell count (RBC, × 10^12^/L), hemoglobin (HGB, g/L), hematocrit (HCT, %), mean corpuscular volume (MCV, fL), mean corpuscular hemoglobin (MCH, pg), mean corpuscular hemoglobin concentration (MCHC, g/L), white blood cell counts (WBC, × 10^9^/L), lymphocyte percentage (LYM %), absolute lymphocyte count (LYM #, × 10^9^/L), blood platelet (PLT, × 10^9^/L), mean platelet volume (MPV, fL), plateletcrit (PCT, fL), and platelet distribution width (PDW, fL). Blood biochemical indices were measured using an automatic biochemical analyzer (Model 7060; Hitachi Ltd., Japan), including glutamate pyruvate transaminase (ALT, U/L), glutamic oxalacetic transaminase (AST, U/L), total bilirubin (TBIL, µmol/L), direct bilirubin (DBIL, µmol/L), blood urea nitrogen (BUN, mmol/L), and creatinine (CREA, µmol/L). AST/ALT ratio and indirect bilirubin (IBIL, µmol/L) were calculated indices. The Lake Louise score (LLS) system scores (Calbet et al. 2002) were also collected in two phases. The LLS questionnaire consists of five items: headache, dizziness, gastrointestinal symptoms, fatigue/weakness, and difficulty sleeping. Each item was rated on a four-point scale (0 = not at all, 1 = mild, 2 = moderate, and 3 = severe). Single item scores are added up, and the maximal score is 15.

### Exploring the Relationship of Phenotypes by Spectral Clustering

**Fig. 2:**
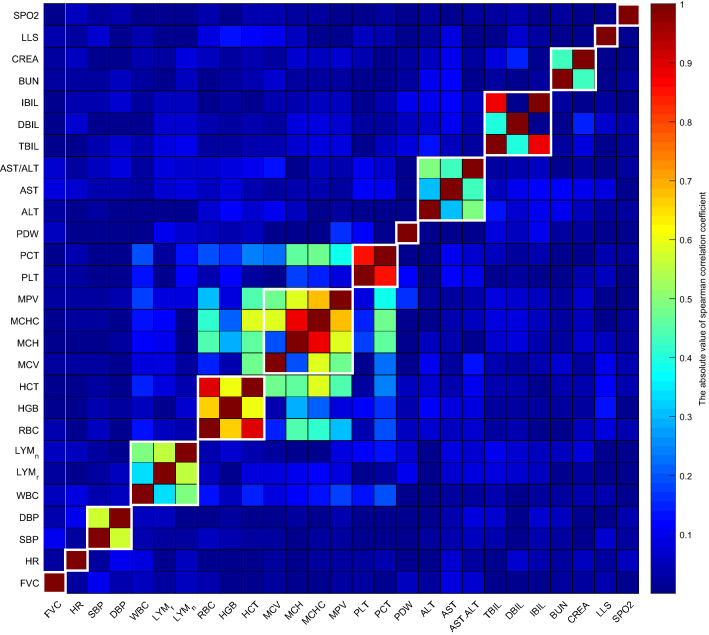
The absolute value of spearman correlation heatmap of 28 physiological phenotypes. The spearman correlation coefficient ranges from 0 (dark blue) to 1 (dark red). The spectral clustering results are marked by white boxes. For example, SBP and DBP are grouped together, and their absolute spearman correlation coefficient is about 0.6 (yellow–green color)

The longitudinal data of high-altitude acclimatization were firstly transformed into change data (Fitzmaurice et al. 2012). All the 28 physiological traits have significant (under Bonferroni correction (Goeman and Solari 2014) changes from baseline to chronic phase at 4300-m highland. In addition, the *p* values were calculated by the Wilcoxon Rank-Sum Test (Wilcoxon 1945) (Table 1). Based on the change data of high-altitude acclimatization, spectral clustering (Hastie et al. 2009; Luxburg 2007) was applied. The similarity matrixes in this study were the absolute values of spearman correlation coefficient (Well and Myers 2003) matrixes of 28 physiological changes from baseline to chronic phase for high-altitude acclimatization. The affinity matrixes were computed by applying a k-nearest neighbor filter (Altman 1992) to build a representation of a graph connecting just the closest dataset points. To compute the graph Laplacian matrix, there was also a need to get the degree matrix, where each diagonal value is the degree of the respective vertex, and all other positions are zero (Luxburg 2007). To determine the number of clusters, the one with maximum eigenvalue gap (Supplementary Fig. 1) was selected (Zelnik-Manor and Perona 2005). The correlation heatmap (Fig. 2) showed the spectral clustering results of 28 physiological phenotypes, and these were clustered into 14 groups (i.e., composite phenotype structure, Fig. 2). The spectral clustering results were the composite phenotype structure (Figs. 1 and 2).

**Table 1:** The 28 physiological traits from 883 Chinese Han young males at baseline and chronic phases of high-altitude acclimatization

Variables	Baseline		Chronic		*p* value*
	Mean	SD	Mean	SD	
ALT	19.11	8.14	15.02	9.08	**6.28E–45**
AST	15.64	5.83	46.05	15.24	**2.33E–139**
AST/ALT	0.85	0.18	3.91	2.54	**2.14E–139**
TBIL	11.94	1.77	14.51	24.90	**1.41E–10**
DBIL	2.69	0.61	5.70	1.70	**3.72E–136**
IBIL	9.25	1.24	8.81	24.95	**1.96E–39**
BUN	5.08	1.12	6.21	1.17	**3.20E–87**
CREA	58.32	10.38	113.02	12.20	**2.85E–139**
WBC	6.21	1.34	8.21	1.70	**1.55E–125**
LYM%	36.16	7.48	40.61	10.71	**7.71E–42**
LYM#	2.21	0.52	3.31	0.99	**1.78E–106**
RBC	4.88	0.39	5.73	0.48	**5.25E–144**
HGB	150.15	10.05	179.59	13.46	**2.07E–144**
HCT	0.44	0.03	0.50	0.04	**1.35E–136**
MCV	90.60	5.14	87.39	4.89	**6.49E–126**
MCH	30.91	2.34	31.39	2.18	**1.16E–21**
MCHC	341.07	17.94	359.14	16.24	**3.97E–85**
PLT	206.88	42.79	258.94	51.49	**2.18E–124**
PCT	2.01	0.42	2.72	0.51	**9.95E–139**
MPV	9.78	1.26	10.54	0.62	**9.59E–68**
PDW	13.79	2.25	18.03	2.53	**9.71E–142**
FVC	444.45	38.26	412.31	59.16	**2.03E–51**
SBP	110.88	10.45	124.34	12.94	**2.51E–87**
DBP	73.21	8.65	75.98	9.61	**7.46E–13**
HR	66.49	9.57	87.16	10.99	**3.24E–123**
Body temperature	36.22	0.12	36.38	0.29	**1.75E–39**
SPO_2_	97.76	2.08	85.82	3.80	**6.64E–132**
LLS	0.88	1.59	1.40	1.73	**1.14E–10**

^*^p values were calculated by Wilcoxon Rank-Sum Test (paired = true)

The significant (under Bonferroni correction) *p* values were shown in bold

### Defining Composite Phenotypes by PLSPM

**Fig. 3:**
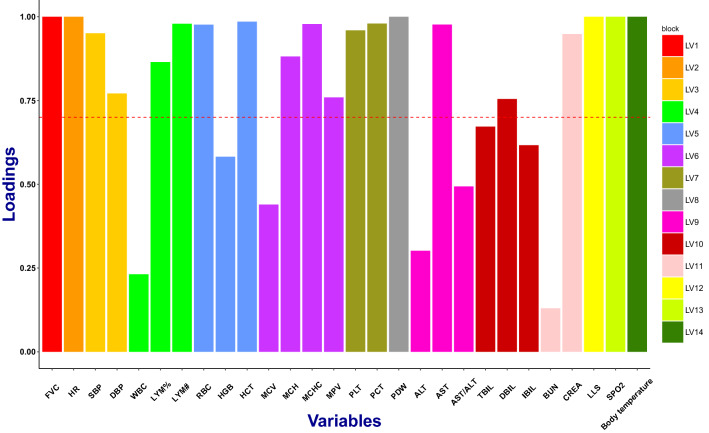
The PLSPM loadings of 14 composite phenotypes of high-altitude acclimatization. The 14 composite phenotypes (LV1, LV2… LV14) are represented by 14 different colors, and the height of each colorful bar is the loading (correlation) of each composite phenotype. Acceptable values for the loadings are values greater than 0.7 (threshold line), indicating that more than 49% (0.7×0.7) of the variability in a single phenotype (like SBP or DBP) is captured by its composite phenotype (like LV3)

Based on the composite phenotype structure, PLSPM (Sanchez 2013; Tenenhaus et al. 2005) was further applied to construct composite phenotypes. Latent variable scores (Sanchez 2013; Esposito Vinzi et al. 2010) were calculated to estimate these composite phenotypes. Since the 28 physiological traits were clustered as 14 groups, there were also 14 composite phenotypes (LV1, LV2… LV14) accordingly. PLSPM is claimed to explain at best the residual variance of the latent variables and potentially also of the manifest variables in any regression run in the model without strong assumptions (Esposito Vinzi et al. 2010). To check the unidimensionality of PLSPM blocks, Cronbach’s alpha, Dillon–Goldstein’s rho and the first eigenvalue of the indicators’ correlation matrix were calculated (Sanchez 2013; Esposito Vinzi et al. 2010). Dillon–Goldstein’s rho focuses on the variance of the sum of variables in the block of latent variable (Sanchez 2013; Esposito Vinzi et al. 2010). Each composite phenotype captures a specific aspect of high-altitude acclimatization (Table 2, Fig. 3 and Supplementary Fig. 2).

**Table 2:** PLSPM composite phenotypes unidimensionality evaluation

	Biological meanings	Manifest variables	Mode	MVs	C.alpha	DG.rho	eig.1st	eig.2nd
LV1	Forced vital capacity	FVC	A	1	1.00	1.00	1.00	0.00
LV2	Heart rate	HR	A	1	1.00	1.00	1.00	0.00
LV3	Blood pressure	SBP, DBP	A	2	0.70	0.87	1.54	0.46
LV4	Immune system	LYM#, LYM%, WBC	A	3	0.55	0.77	1.76	1.21
LV5	Number of red cells	RBC, HCT, HGB	A	3	0.89	0.93	2.45	0.48
LV6	Hemoglobin concentration	MCH, MCHC, MPV, MCV	A	4	0.79	0.87	2.52	1.01
LV7	Number of platelets	PLT, PCT	A	2	0.94	0.97	1.88	0.12
LV8	Platelet distribution width	PDW	A	1	1.00	1.00	1.00	0.00
LV9	Liver function	ALT, AST, AST/ALT	A	3	0.25	0.04	1.35	1.31
LV10	Bilirubin	TBIL, DBIL, IBIL	A	3	0.59	0.79	2.00	1.00
LV11	Renal function	BUN, CREA	A	2	0.61	0.84	1.44	0.56
LV12	Lake Louise score	LLS	A	1	1.00	1.00	1.00	0.00
LV13	Oxygen saturation	SPO_2_	A	1	1.00	1.00	1.00	0.00
LV14	Body temperature	Body temperature	A	1	1.00	1.00	1.00	0.00

Overall 14 composite phenotypes are shown as the latent variables (LV1, LV2…LV13, LV14)

### Revealing Physiological Heterogeneity by k-Means

**Fig. 4:**
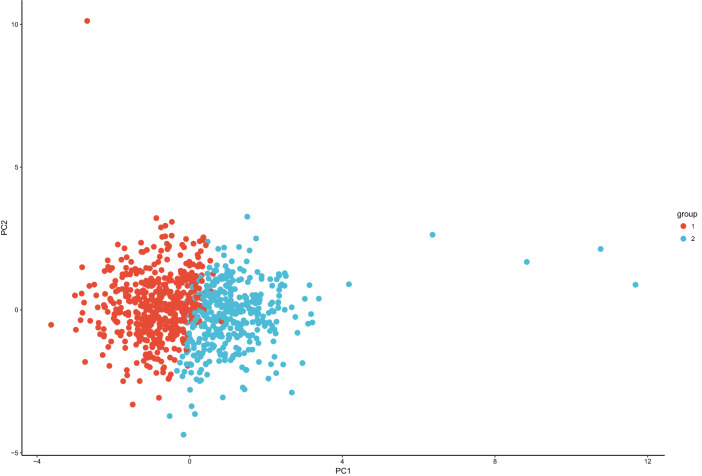
k-Means clustering results on individuals using the 14 composite phenotypes (LVs). The 883 individuals are clustered into two groups (group 1 with 508 individuals and group 2 with 375 individuals) based on their composite phenotype scores. The PCA plot is the just visualization of k-means clustering results (group 1 with red color, and group 2 with blue color accordingly)

**Fig. 5:**
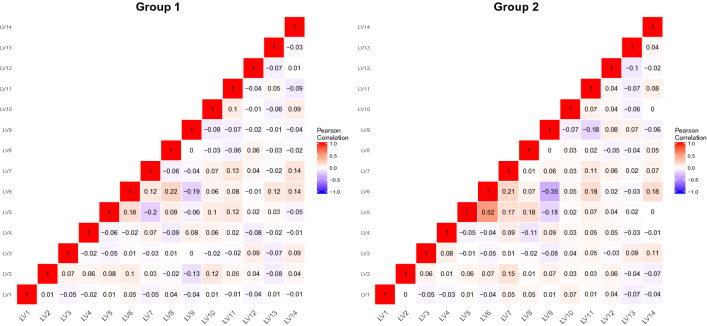
The pairwise Pearson correlation heatmap of 14 composite phenotypes (LV1, LV2… LV14) of two groups. The Pearson correlation coefficient ranges from − 1 (blue) to 1 (red). The left figure represents the Pearson correlation heatmap of 14 LVs of group 1, and the right figure represents the Pearson correlation heatmap of 14 LVs of group 2

Based on the 14 composite phenotypes, k-means clustering was applied to explore physiological heterogeneity in high-altitude acclimatization (Fig. 4). The optimal number of clusters is 2 (Supplementary Fig. 3) following the majority rule of 26 indices (Charrad et al. 2012). The silhouette plot (Supplementary Fig. 4) for k-means clustering also showed that observations were well clustered (Rousseeuw 1987). Thus, the 883 Chinese Han young males were clustered into two groups (group 1 with 508 individuals and group 2 with 375 individuals, Fig. 4) based on the 14 composite phenotypes of high-altitude acclimatization. To further investigate the physiological patterns of the two groups, a pairwise Pearson correlation (Pearson 1895) heatmap was generated (Fig. 5).

### Modeling Oxygen Saturation Variation by Multivariate Linear Regression

To model how physiological traits are systematically related to SpO_2_ changes in a high-altitude acclimatization process, two multivariate linear regression models (Freedman 2009) were constructed. Model 1 is constructed by original 28 physiological traits changes from baseline to chronic phase at 4300-m highland, and SpO_2_ is the dependent variable (*Y*). Model 2 is constructed by 13 composite phenotypes (excluding LV13, i.e., SpO_2_) of high-altitude acclimatization, and SpO_2_ remained the dependent variable (*Y*). To evaluate the fitness of the two models, Akaike information criterion (AIC) (Akaike 1998; Aho et al. 2014), Bayesian information criterion (BIC) (Aho et al. 2014; Schwarz 1978), tenfold cross-validation (CV) (Kohavi 1995), root-mean-square error (RMSE) (Hyndman and Koehler 2006), and leave-one-out RMSE were measured (Table 3). We also employed the Wilcoxon signed-rank test (Wilcoxon 1945) to compare the tenfold CV MSE and leave-one-out MSE of the two models (Models 1 and 2).

**Table 3:**
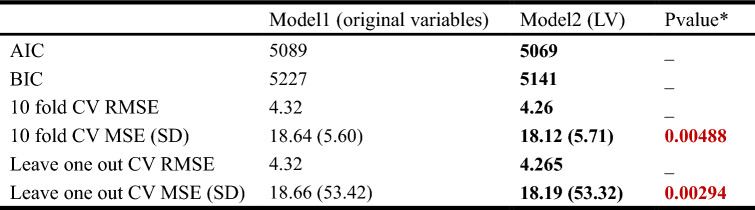
Evaluation the goodness of fit of two multivariate linear regression models

The measurement indices with better fitness were shown in bold

The significant *p* values (*p* value < 0.05) were marked as bold and red color

^*^Tenfold cross-validation MSE and leave-one-out MSE of two models were calculated, and Wilcoxon signed-rank test was employed to test for significant difference, and alternative hypothesis (Model 1–Model 2): true location is greater than 0

All the computation process of this study was realized by R (v3.3.1) (Team RC 2014), and the related figures were generated with Matlab (R2015b) (Incorporation 2005), ‘ggplot2′ (Wickham 2016) and ‘igraph’ (Csardi and Nepusz 2006) R packages. The computation process of composite phenotype scores was completed with the ‘plspm’ (Sanchez 2013) R package. *k*-Means clustering was completed with the ‘factoextra’ (Kassambara and Mundt 2016) and ‘NbClust’ (Charrad et al. 2012) R packages. The multivariate linear regression models were calculated using the ‘stats’ R package.

## Results

### Exploring the Correlation Between Phenotypes and High-Altitude Acclimatization

In this study, we collected the 28 physiological traits from 883 Chinese Han young males at the baseline phase of living at a location with an altitude of 50 m in China before going to highland and the chronic phase (living at highland of above 4300 m in China for about 1 month) of altitude acclimatization (Table 1). All the 28 physiological traits show significant (Bonferroni correction) changes from the baseline to chronic phase at 4300-m highland (Table 1). These results indicate that a series of physiological phenotypes are involved in high-altitude acclimatization process (West et al. 2012; Muza et al. 2010; Peng et al. 2013). Since we are mainly concerned with changes in these phenotypes, the longitudinal data were transformed into change data (Fitzmaurice et al. 2012) using Measure_chronic-baseline_ = Measure _chronic_—Measure_baseline_. These data were employed in the subsequent analyses.

By analyzing the correlation between pairwise phenotypes, we found that the phenotypes are structured (Fig. 2). For example, RBC, HGB and HCT have a strong correlation with each other, and RBC has almost no correlation with LLS and SPO_2_. To further explore the relationship of phenotypes, the spectral clustering algorithm (Hastie et al. 2009; Luxburg 2007) was applied to group these 28 physiological phenotypes. The correlation heatmap (Fig. 2) showed the spectral clustering results of 28 physiological phenotypes, which were clustered into 14 groups (i.e., composite phenotype structure, Fig. 2) (Zelnik-Manor and Perona 2005).

### Defining Composite Phenotypes of High-Altitude Acclimatization

Based on the revealed aforementioned structure, PLSPM (Sanchez 2013; Tenenhaus et al. 2005; Esposito Vinzi et al. 2010) was applied to extract composite phenotypes of high-altitude acclimatization. Overall, 14 composite phenotypes (LVs) were extracted as the latent variables (Sanchez 2013). Each composite phenotype is a linear combination of their manifest variables (Tenenhaus et al. 2005) and captures a specific aspect of high-altitude acclimatization (Fig. 3, Table 2 and Supplementary Fig. 2). The LV5 explained the variance of RBC, HCT, and HGB, which mainly reflect the number of red cells (Dillon–Goldstein’s rho = 0.93, Table 2 and Fig. 3). The LV6 explained the variance of MCH, MCHC, MPV, and MCV, which reflect hemoglobin levels. As changes in MCH and MCHC were negatively correlated with MPV and MCV, we changed both MCH and MCHC signs (multiplied by − 1) to maintain positive loadings (Sanchez 2013). The LV12 is equivalent to single-phenotype LLS, and the LV13 represents single-phenotype SPO_2_.

### Revealing Physiological Heterogeneity in High-Altitude Acclimatization

To explore physiological heterogeneity in high-altitude acclimatization, we applied k-means clustering algorithm (Hastie et al. 2009; Luncien et al. 1967) using the 14 composite phenotypes. Thus, the 883 individuals could be clustered into two groups (group 1 with 508 individuals and group 2 with 375 individuals, Fig. 4, Supplementary Figs. 4 and 5) based on the 14 composite phenotypes of high-altitude acclimatization. The separation of two groups of the individuals is mainly contributed by hemoglobin concentration (LV6, Wilcoxon Rank-Sum Test’s *p* value = $3.36\times {10}^{-90}$), number of red cells (LV5) and platelets (LV7) (Supplementary Table 1 and Supplementary Fig. 5). The results demonstrate physiological heterogeneity in high-altitude acclimatization among these sampled individuals, especially in the phenotypes related to oxygen-carrying capacity (West et al. 2012; Calbet et al. 2002; Vij 2009), including hemoglobin concentration, number of red cells platelet (Supplementary Table 1 and Supplementary Fig. 5). The increase of red cell number and hemoglobin concentration improves the oxygen-carrying capacity of blood to compensate for the reduction in oxygen saturation (West et al. 2012; Hackett et al. 1985; La 1988).

To further characterize the relationship of the 14 composite phenotypes in each group, we calculated the pairwise Pearson correlation (Pearson 1895, Fig. 5). For instance, significant correlation (Pearson’s *r* = 0.12, *p* value = 0.006, Supplementary Table 2) was found between LV6 and LV13 in group 1, but not in group 2 (Pearson’s *r* = − 0.03, *p* value = 0.51, Supplementary Table 3). To compare the difference of these two Pearson correlation coefficients, Fisher’s z transformation (Fisher 1921, 1992; Cohen et al. 2017; Diedenhofen and Musch 2015) was applied (*p* value = 0.02, Supplementary Table 4). There is a negative correlation (Pearson’s *r* = − 0.2) between LV5 and LV7 in group 1, whereas the positive correlation (Pearson’s *r* = 0.17, Fisher’s z transformation *p* value = $5.58 \times {10}^{-8}$) was observed between them in group 2. Thus, we can compare the correlation networks of multiple physiological traits intuitively and focus on composite phenotypes instead of their manifest variables.

### Modeling Oxygen Saturation Variation of High-Altitude Acclimatization

Oxygen saturation (SpO_2_) reflects the most straightforward physiological change during high-altitude acclimatization (Muza et al. 2010; West 2004, 2017; Martin et al. 2014). To model how other physiological traits systematically relate to SpO_2_ during high-altitude acclimatization, we constructed two multivariate linear regression models. Model 1 was constructed by the original 28 physiological traits changes from the baseline to the chronic phase at 4300-m highland, and SpO_2_ is the dependent variable (Y). To compare with this model, Model 2 was constructed by 13 composite phenotypes (excluding LV13, i.e., SpO_2_) of high-altitude acclimatization.

To evaluate the goodness of fit between the two models, the AIC (Akaike 1998; Aho et al. 2014), Bayesian information criterion (BIC) (Aho et al. 2014; Schwarz 1978), tenfold cross-validation (CV) (Kohavi 1995), root-mean-square error (RMSE) (Hyndman and Koehler 2006) and leave-one-out RMSE were measured (Table 3). Model 2 showed better fitness than Model 1 in all measurement indices (Table 3), suggesting that the composite phenotypes are better performed in capturing the variation of high-altitude acclimation. From the multivariate regression result of Model 2, we also found that LV12 (LLS) is the most significant ($\beta =- 0.29, p\mathrm{ value}=0.04,$ Supplementary Table 5) trait that influences SpO_2_. SpO_2_ has been well studied as the predictor/indicator of AMS or LLS (West et al. 2012; Muza et al. 2010; West 2004; Brierley et al. 2012; Burtscher et al. 2008; Karinen et al. 2010; Koehle et al. 2010). Individuals who successfully maintained their oxygen saturation at rest were most likely not to develop AMS (Muza et al. 2010; West 2004; Karinen et al. 2010).

## Discussion

In this study, we developed a data-driven strategy (Fig. 1) to extract composite phenotypes from multiple physiological phenotypes of high-altitude acclimatization in a large-scale Chinese Han longitudinal cohort. We firstly explored the relationship among the phenotypes of high-altitude acclimatization. Then, we extracted 14 composite phenotypes from 28 physiological traits changes of high-altitude acclimatization. This strategy could be applied to other complex traits, for example, immune diseases, cardio metabolic traits or other complex diseases.

Altitude acclimatization comprises a number of physiological responses to mitigate the effects of hypoxia (West et al. 2012; West 2004). There are various methods to analyze the longitudinal data such as linear mixed models (Verbeke 1997) and data transformation (Fitzmaurice et al. 2012). Since we are mainly concerned on changes in these phenotypes, the transforming of the longitudinal data into change data is also a promising alternative (Muza et al. 2010; Peng et al. 2013; Fitzmaurice et al. 2012; Richalet et al. 2012). Thus, the transformed data (Measure_chronic-baseline_ = Measure _chronic_—Measure _baseline_) were used in this study. In addition, there were only two time points (baseline and chronic) in this study, and the data transformation is a direct and effective method considering the temporal information.

The most important physiological responses of altitude acclimatization are in the cardiorespiratory and the hematology system (Muza et al. 2010; West 2004, 2017; Martin et al. 2014; Peacock and Jones 1997). SpO_2_ reflects the most straightforward physiological changes. Physiological changes of the hemoglobin concentration in the blood are well known (West et al. 2012; Lundby et al. 2004; Peng et al. 2013; Brierley et al. 2012). Besides the aforementioned phenotypes, we also measured several blood biochemical phenotypes of both kidney and liver function (Table 1) which were important but rarely studied (Wang et al. 2018). We found the significant increase of AST during the altitude acclimatization, indicating potential liver injury (Feng et al. 2011). Moreover, the increase of BUN and CREA reflected the progressive fall in glomerular filtration rate, which suggested the damage of kidney (Ozturk et al. 2007).

Since individual single traits do not effectively reflect the complex mechanism of high-altitude acclimatization (West et al. 2012; West 2004; Peng et al. 2013), the analysis of composite phenotypes could be considered as a promising alternative (Inglese et al. 2017; Ried et al. 2016; Holmes et al. 2008). Among several methods, PLS-based composite phenotypes have relatively interpretable biological meanings (Peng et al. 2013). In particular, PLSPM can be applied to analyze the multiple relationships among the blocks of variables (Sanchez 2013).

Generally, there are two ways to define composite phenotypes in PLSPM framework (Peng et al. 2013; Esposito Vinzi et al. 2010), and one is using the prior specific domain knowledge, while the other is using some data-driven methods such as clustering. Clustering is the task of grouping a set of objects in such a way that objects in the same group are more similar to each other than to those in other groups. In our study, the generalized standard spectral clustering (Hastie et al. 2009; Luxburg 2007) was employed to detect the composite phenotype structure (Fig. 2) for high-altitude acclimatization.

To assess the physiological heterogeneity in high-altitude acclimatization, we applied the k-means clustering algorithm on individuals by using the 14 composite phenotypes. However, k-means clustering may be affected by multicollinearity, and when multiple correlations between variables were above 0.5, the clustering results would be misleading (Sambandam 2003). Whereas, most correlations (Pearson’s r) of the 14 composite phenotypes were < 0.2 (Supplementary Fig. 6). Thus, the characterization of population physiological heterogeneity was not markedly affected. We observed physiological heterogeneity in high-altitude acclimatization of the sampled individuals, especially in the phenotypes related to oxygen-carrying capacity including hemoglobin concentration, number of red cells and platelet (West et al. 2012; Calbet et al. 2002; Vij 2009).

Furthermore, SpO_2_ has been well studied as the predictors/indicators of AMS or LLS (West et al. 2012; Muza et al. 2010; West 2004; Brierley et al. 2012; Burtscher et al. 2008; Karinen et al. 2010; Koehle et al. 2010), and while the studies exploring the relationship between LLS and SpO_2_ in chronic hypoxia are limited. In our study, the oxygen saturation variations were modeled in altitude acclimatization using composite phenotypes, and revealed the association (Table 3 and Supplementary Table 5) with LLS (LV12) and SpO_2_ from the baseline to chronic phase at 4300-m highland. These findings provide the insights into physiological mechanism of chronic hypoxia (Corno et al. 2002).

28 physiological phenotypes were studied on our work covering the respiratory function, cardiac function, oxygen delivery function, hematology, oxygen saturation, kidney function, liver function and LLS. However, there are still phenotypes not involved in this study such as muscle metabolism, oxygen consumption, electrocardiogram, electroencephalogram, and organism metabolism. The data of this study were collected at two time points of high-altitude acclimatization, which may be incomplete. Besides, the subjects in this study were all young males, and the physiological responses of females may vary. More importantly, other factors such as the genetic variations should be further studied to understand the potential physiological mechanism of high-altitude acclimatization (West 2004, 2017).

In summary, we have developed a strategy constituting both spectral clustering and PLSPM to define the composite phenotypes. In addition, we effectively used this strategy to capture 14 composite phenotypes from 28 physiological phenotypes of high-altitude acclimatization. The 14 composite phenotypes have clear meaning in physiology and explain most of the observed variance in statistics. Based on these composite phenotypes, we first observed physiological heterogeneity among individuals in high-altitude acclimatization. In addition, we compared the performance of composite phenotypes and regular phenotypes in predicting the changes of SpO_2_. Both analyses showed that the composite phenotypes is better performed in capturing the variation of high-altitude acclimation. This new strategy of defining and applying composite phenotypes may be potentially employed as a general strategy for studying the complex traits (Wei et al. 2014), particularly in the analysis of phenomics (Houle et al. 2010; Zbuk and Eng 2007).

## Electronic supplementary material

Below is the link to the electronic supplementary material.

Supplementary Fig. 1 The eigenvalue gap of spectral clustering. The eigenvalue gap was maximized to choose the best number of spectral clustering (red line). And the best clustering number is 14

Supplementary Fig. 2 The 14 composite phenotypes of high altitude acclimatization. The numbers on the arrows are the PLSPM loadings of 14 composite phenotypes, which is the same as Figure 3

Supplementary Fig. 3 The optimal number of k-means clustering on individuals. The optimal number of clusters is 2 following the majority rule of total 26 clustering indices

Supplementary Fig. 4 The silhouette plot of k-means clustering on individuals. Silhouette values range from 1 to -1, when silhouette value is close to 1 indicating that the individuals are well clustered. The silhouette plot for k-means clustering showed that observations are well clustered

Supplementary Fig. 5 The histogram plot and density plot of each LV (14 LVs) between 2 groups (group 1 with red color and group 2 with blue color). And we further calculated the Wilcoxon Rank Sum test p value (the title of histogram plot) of each LV between 2 groups.

Supplementary Fig. 6 The pairwise Pearson correlation heatmap of 14 composite phenotypes (LV1, … , LV14). The Pearson correlation coefficient ranges from -1 (blue) to 1(red)

Supplementary file7 (DOCX 15 KB)

Supplementary file8 (DOCX 15 KB)

Supplementary file9 (DOCX 16 KB)

Supplementary file10 (DOCX 15 KB)

Supplementary file11 (PDF 19 KB)
