# Supplementary figures and images for: Using Composite Phenotypes to Reveal Hidden Physiological Heterogeneity in High-Altitude Acclimatization in a Chinese Han Longitudinal Cohort
Source: Phenomics. 2021 Feb 22;1(1):3–14. doi: 10.1007/s43657-020-00005-8 (PMC9584130; doi:10.1007/s43657-020-00005-8)

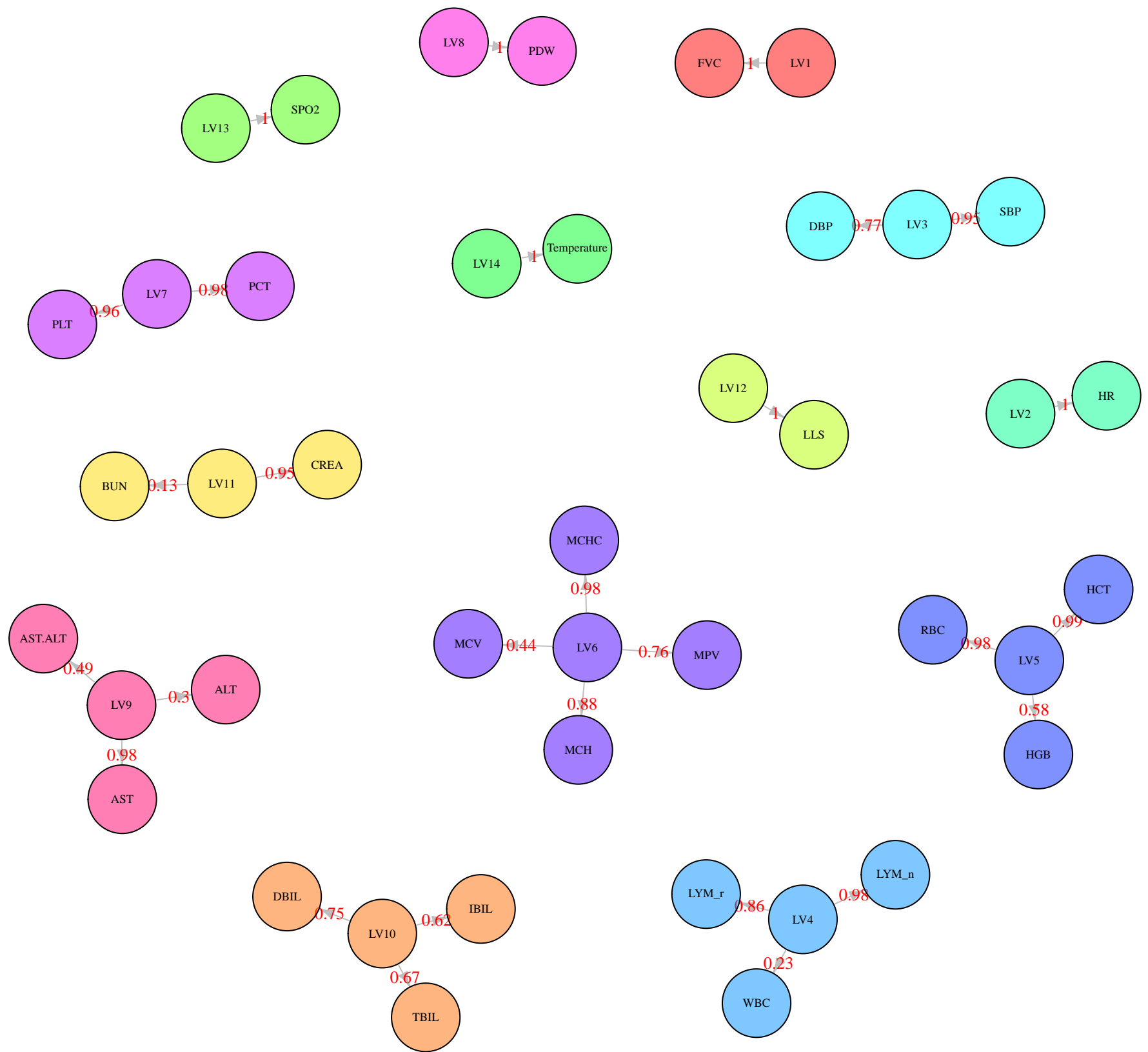

Supplement: Supplementary file 1 — Supplementary Fig. 1 The eigenvalue gap of spectral clustering. The eigenvalue gap was maximized to choose the best number of spectral clustering (red line). And the best clustering number is 14 [file 43657_2020_5_MOESM1_ESM.pdf]

Optimal number of clusters –  $k = 2$

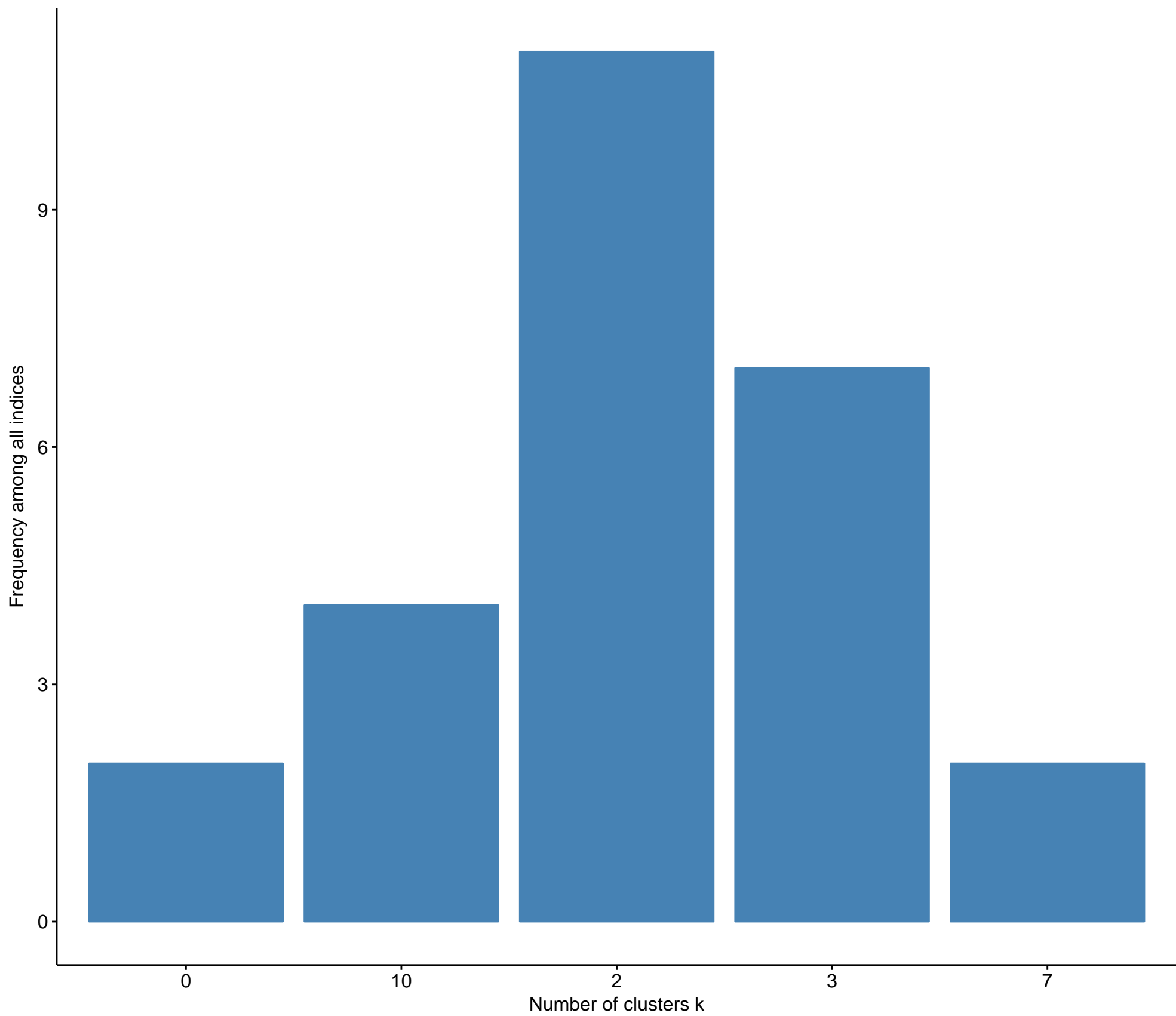

Supplement: Supplementary file 2 — Supplementary Fig. 2 The 14 composite phenotypes of high altitude acclimatization. The numbers on the arrows are the PLSPM loadings of 14 composite phenotypes, which is the same as Figure 3 [file 43657_2020_5_MOESM2_ESM.pdf]

Clusters silhouette plot  
Average silhouette width: 0.08

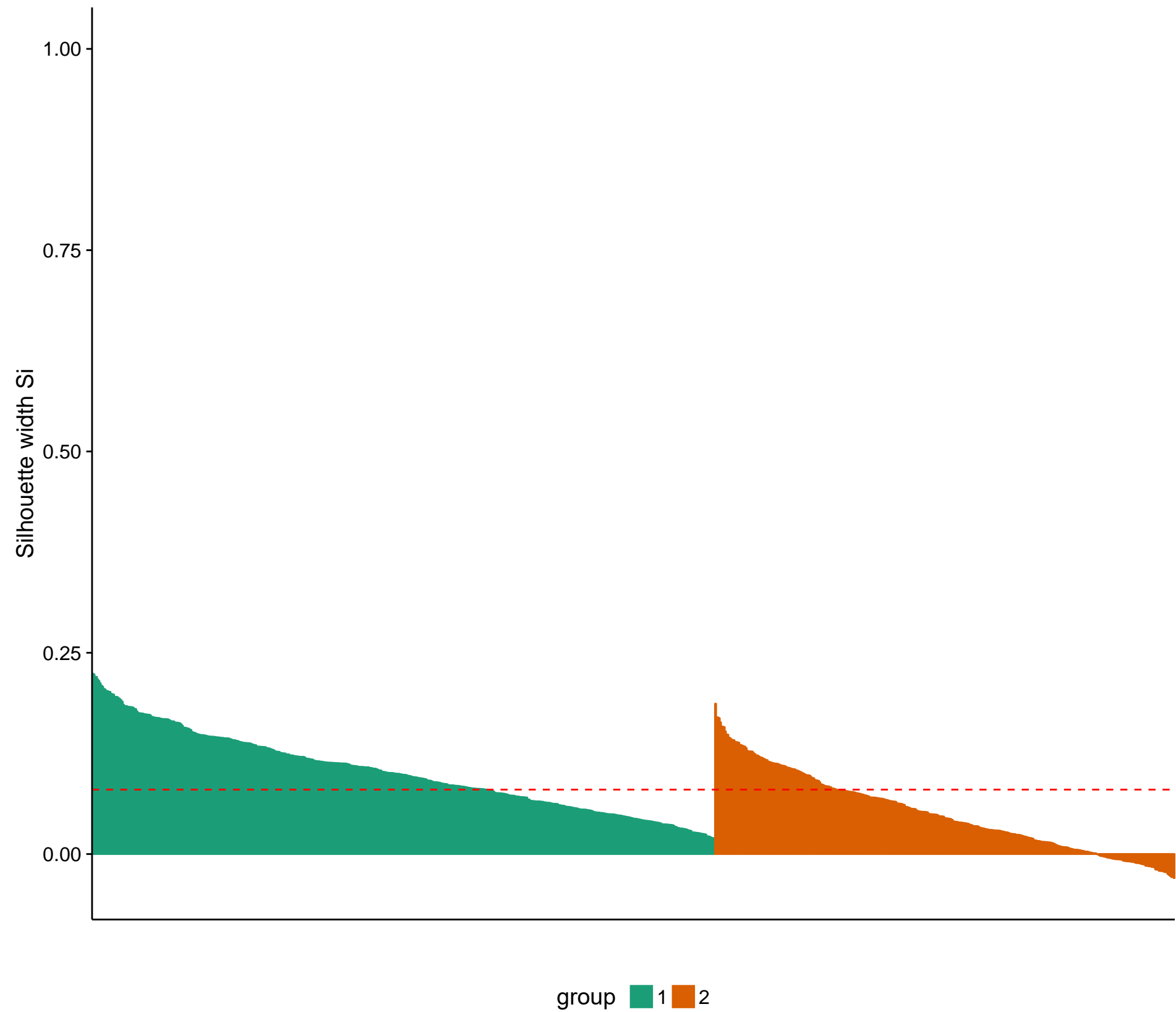

Supplement: Supplementary file 3 — Supplementary Fig. 3 The optimal number of k-means clustering on individuals. The optimal number of clusters is 2 following the majority rule of total 26 clustering indices [file 43657_2020_5_MOESM3_ESM.pdf]

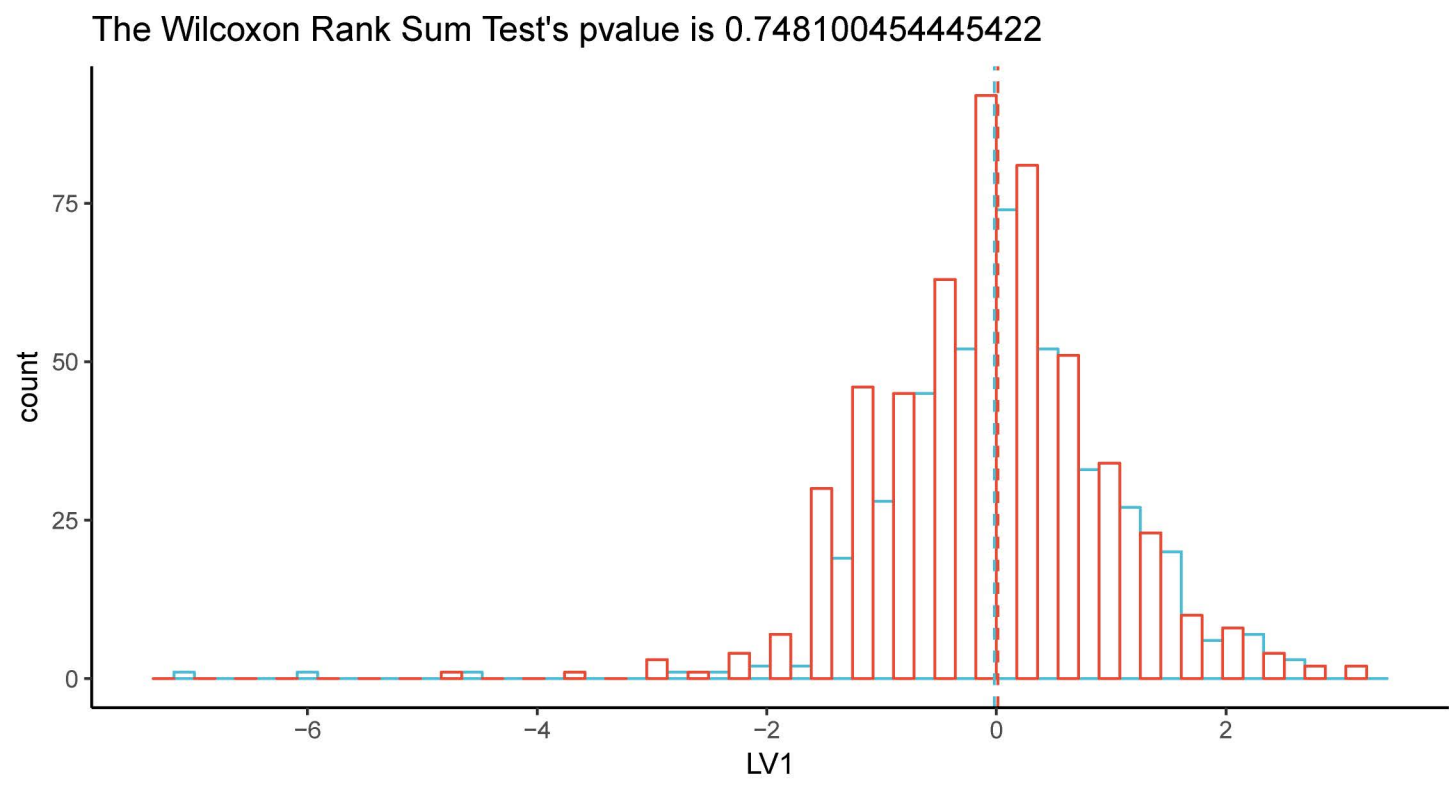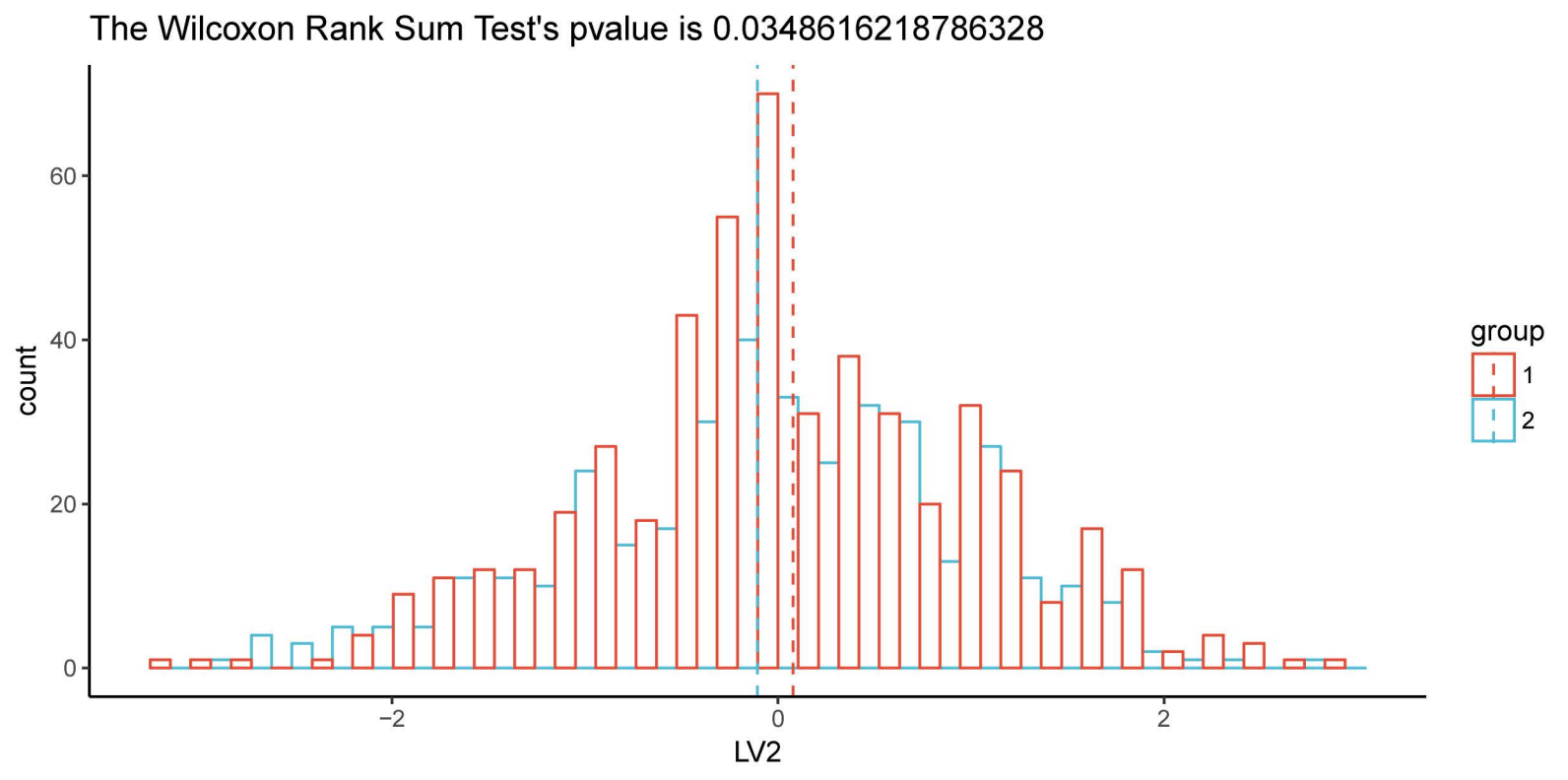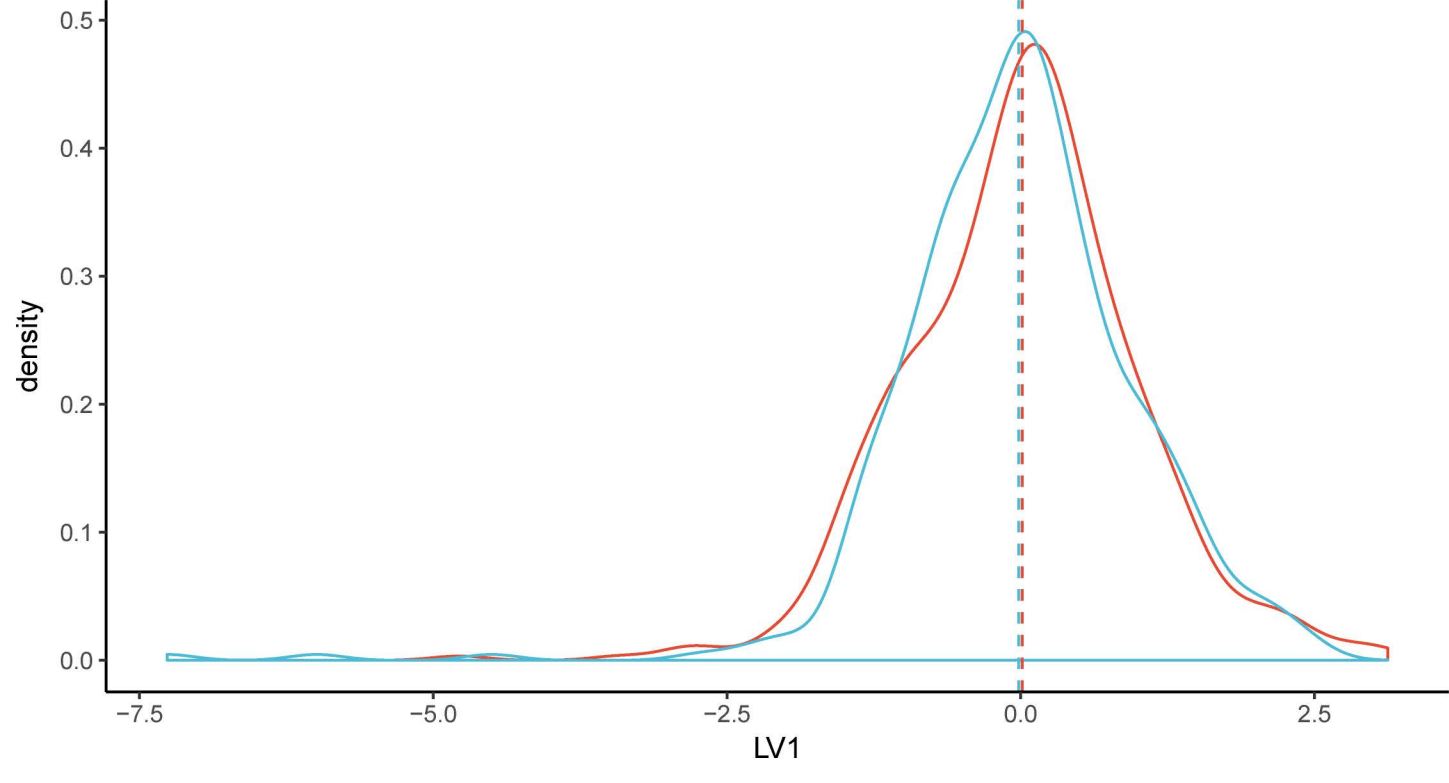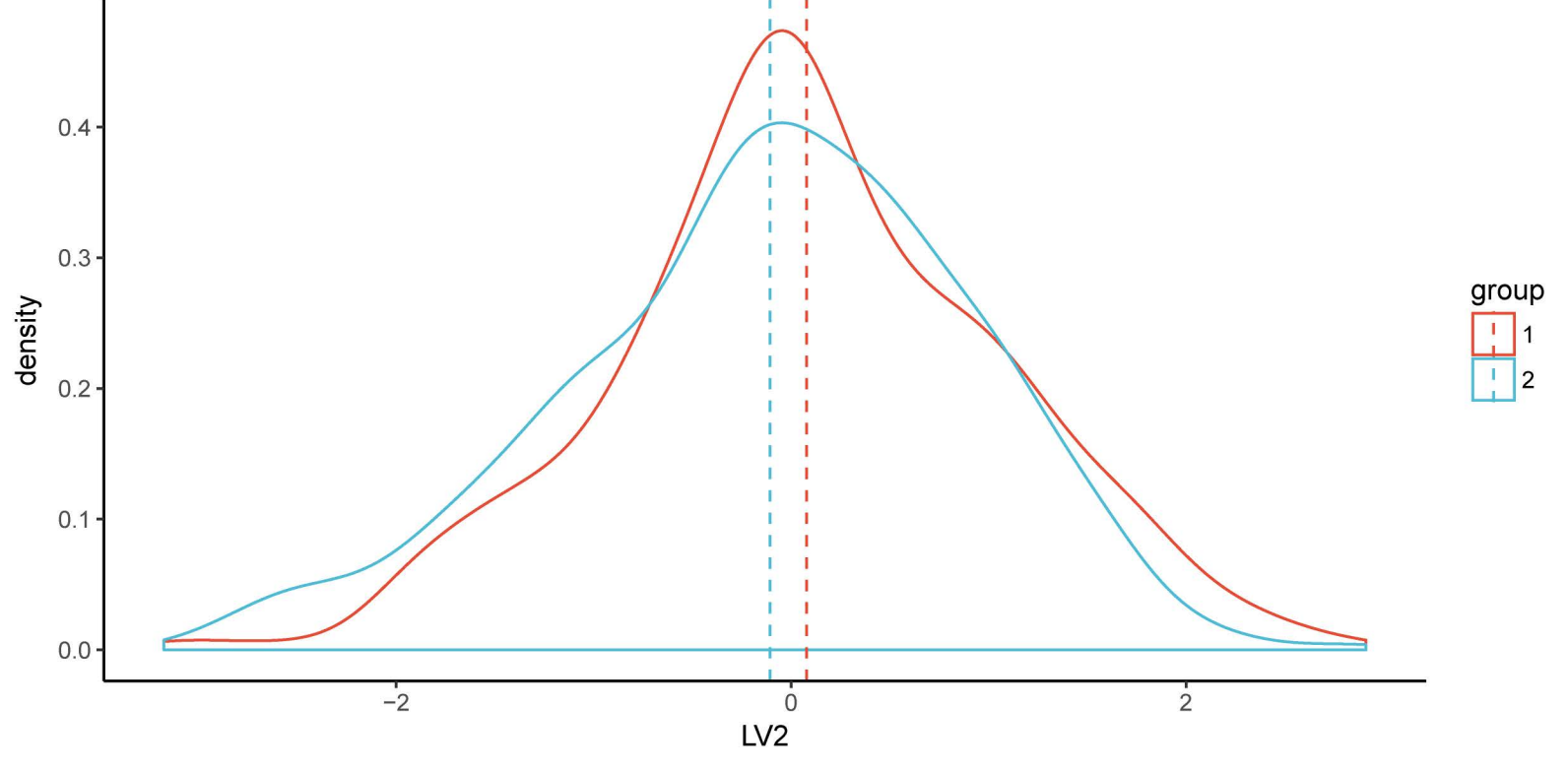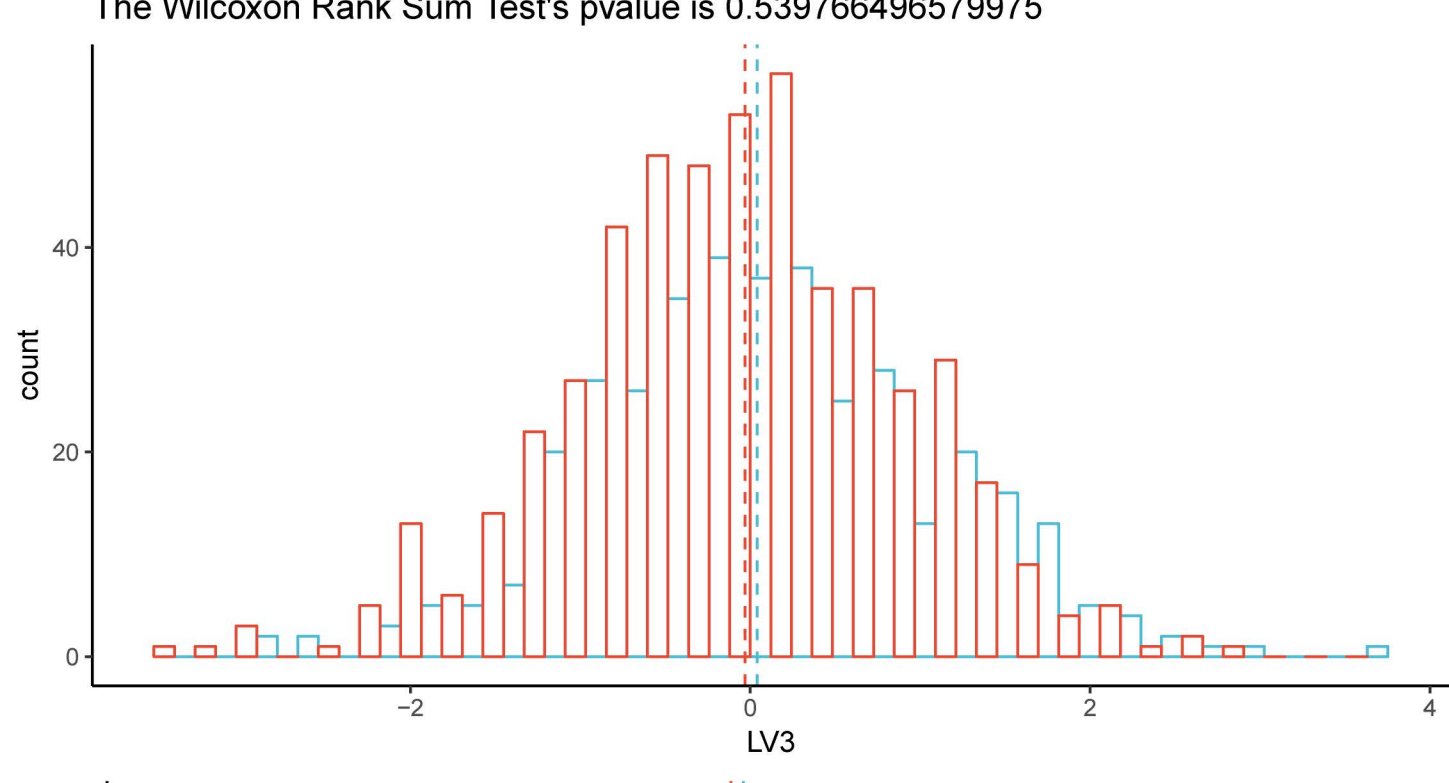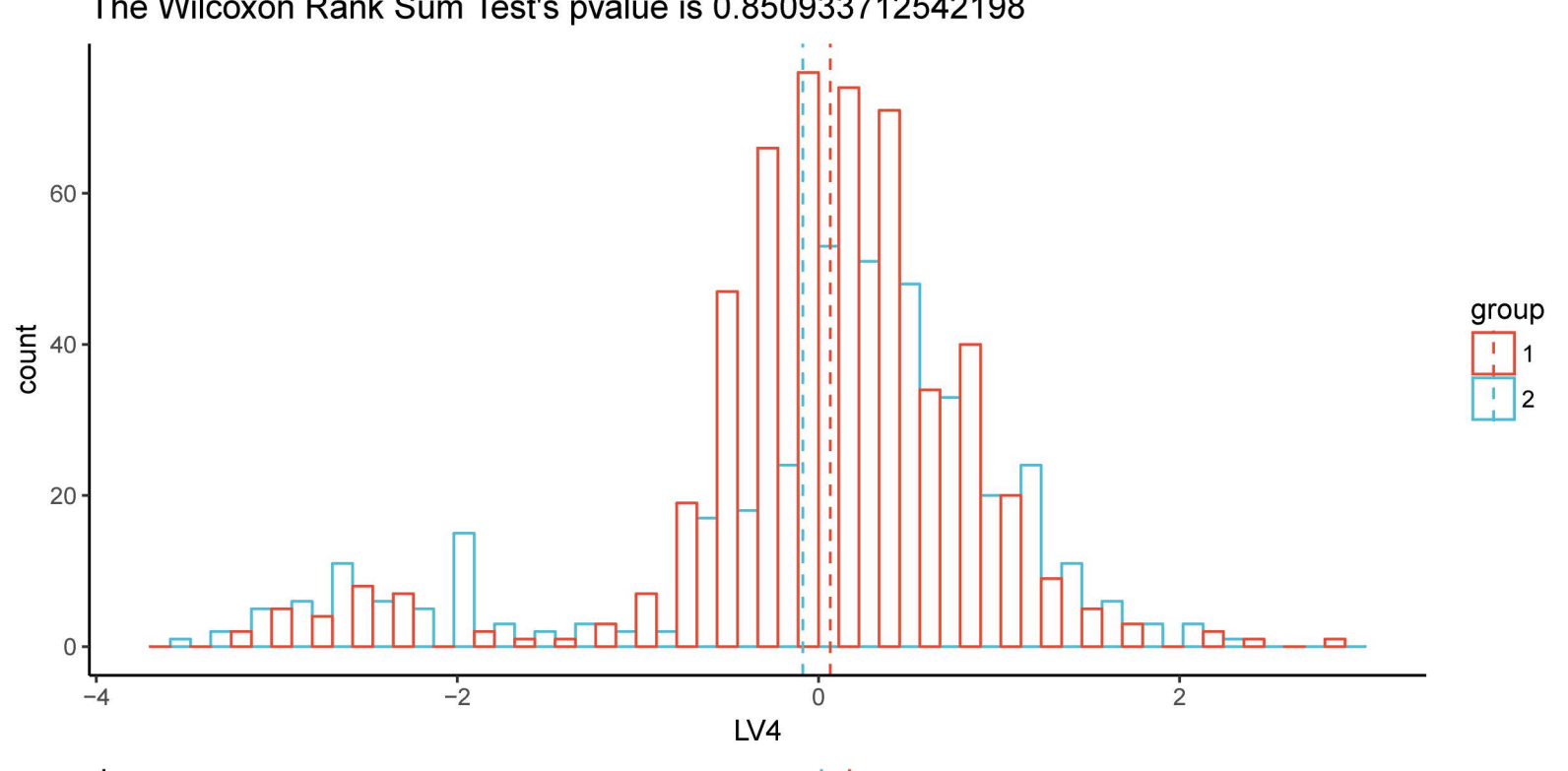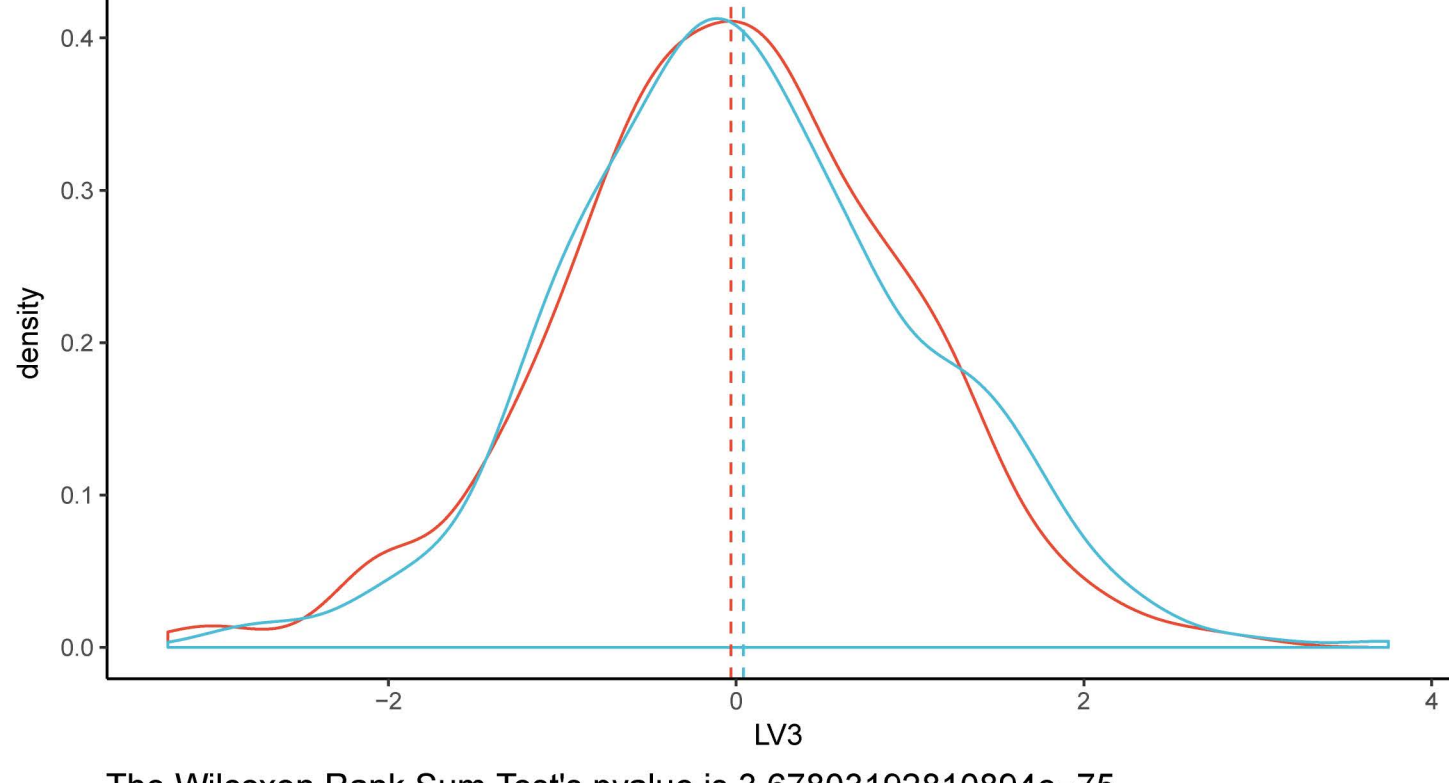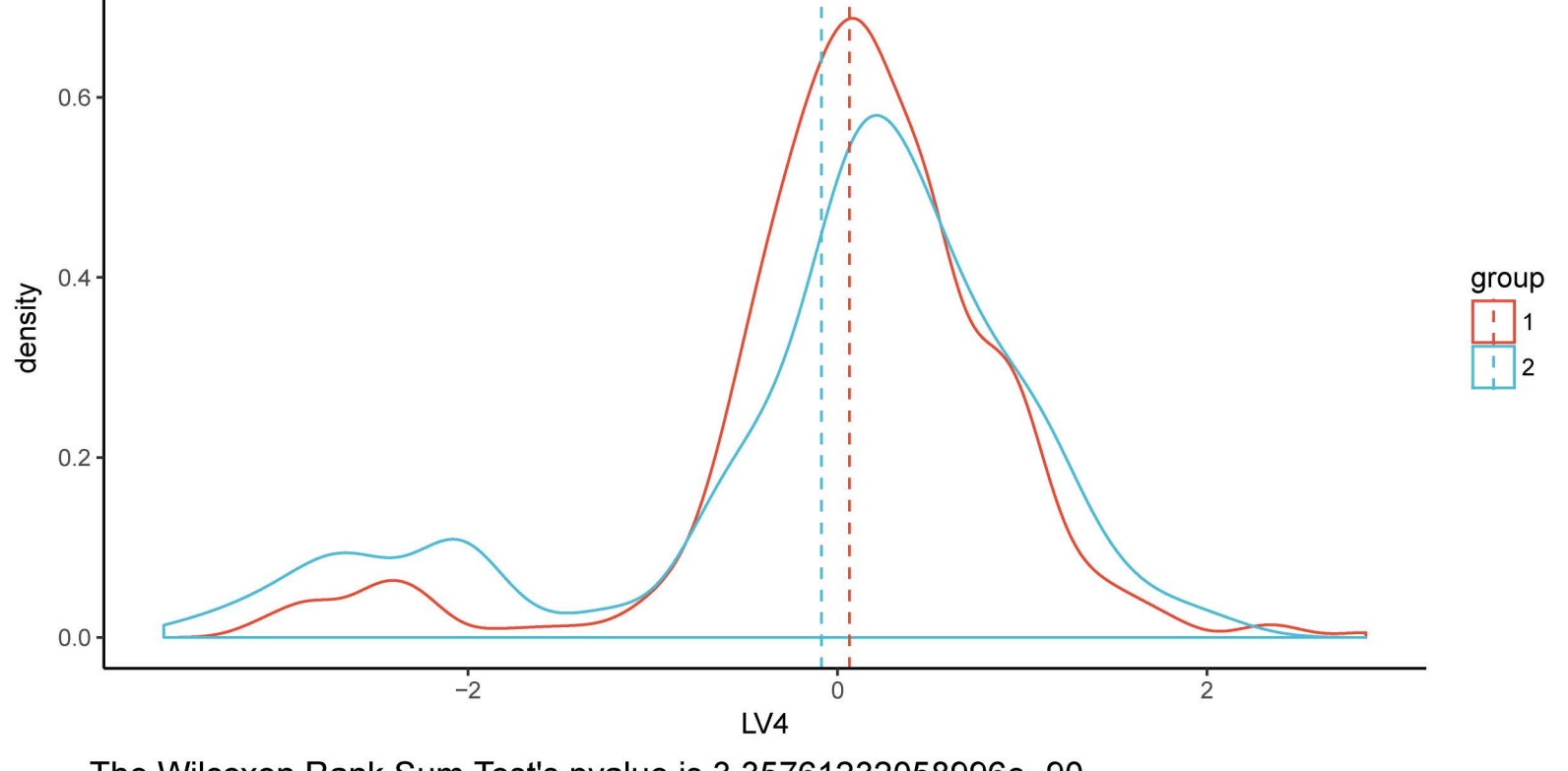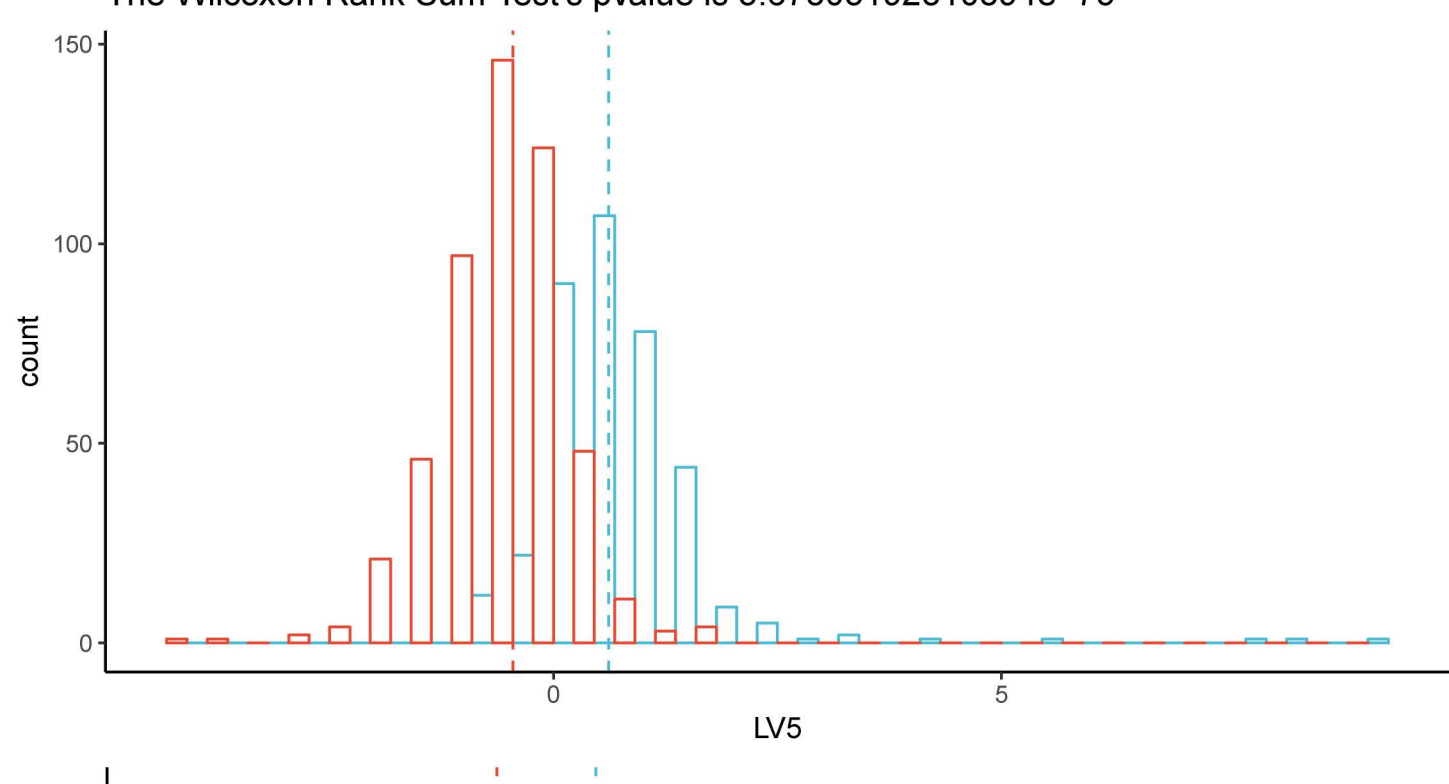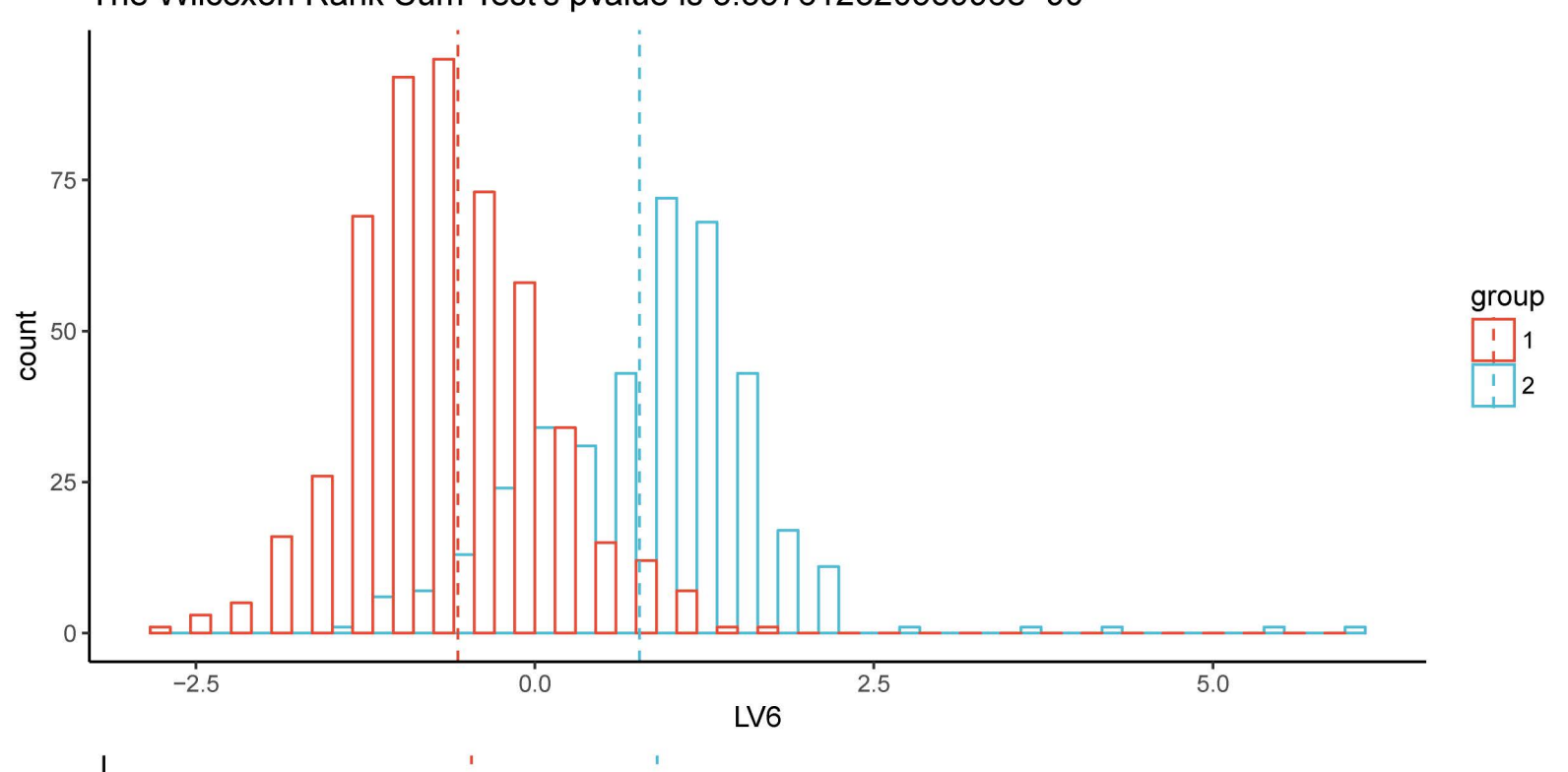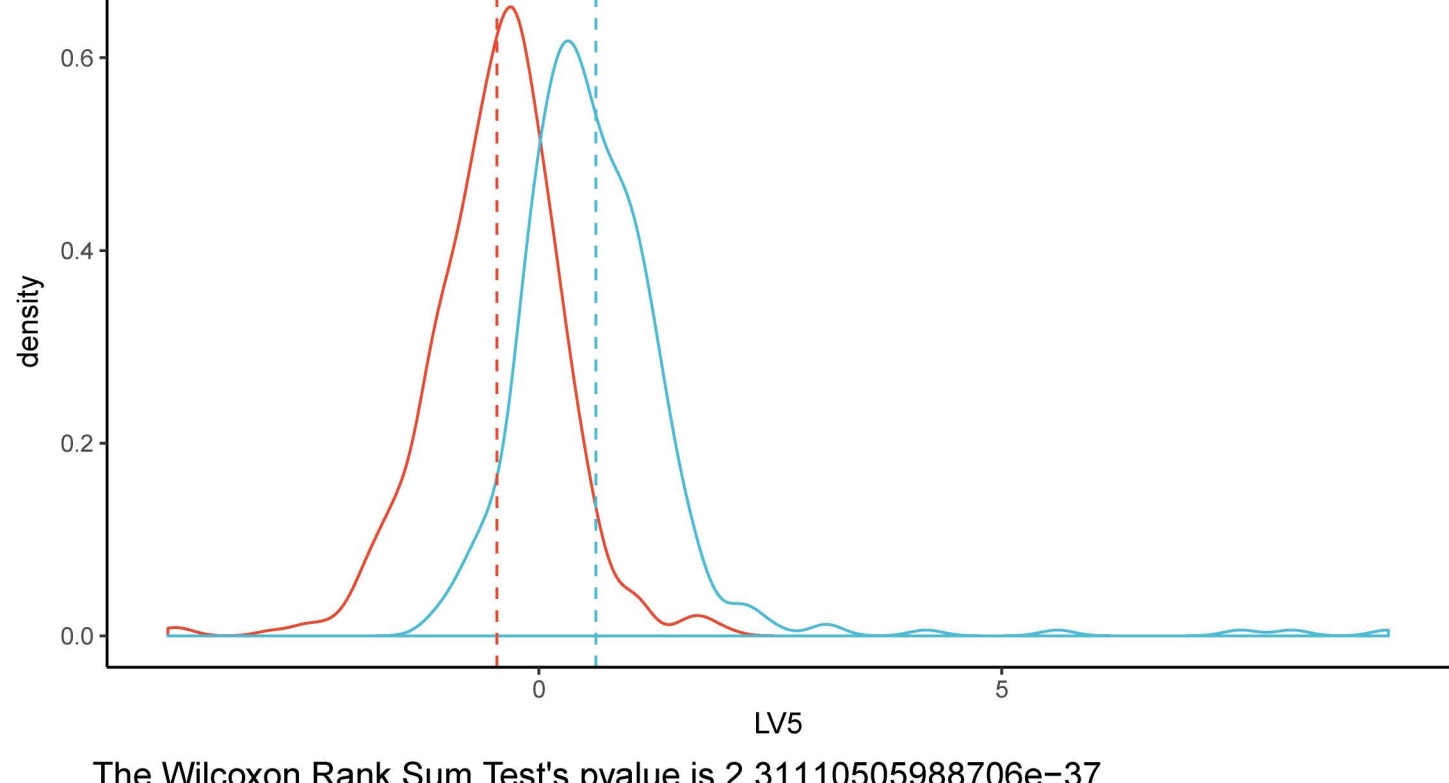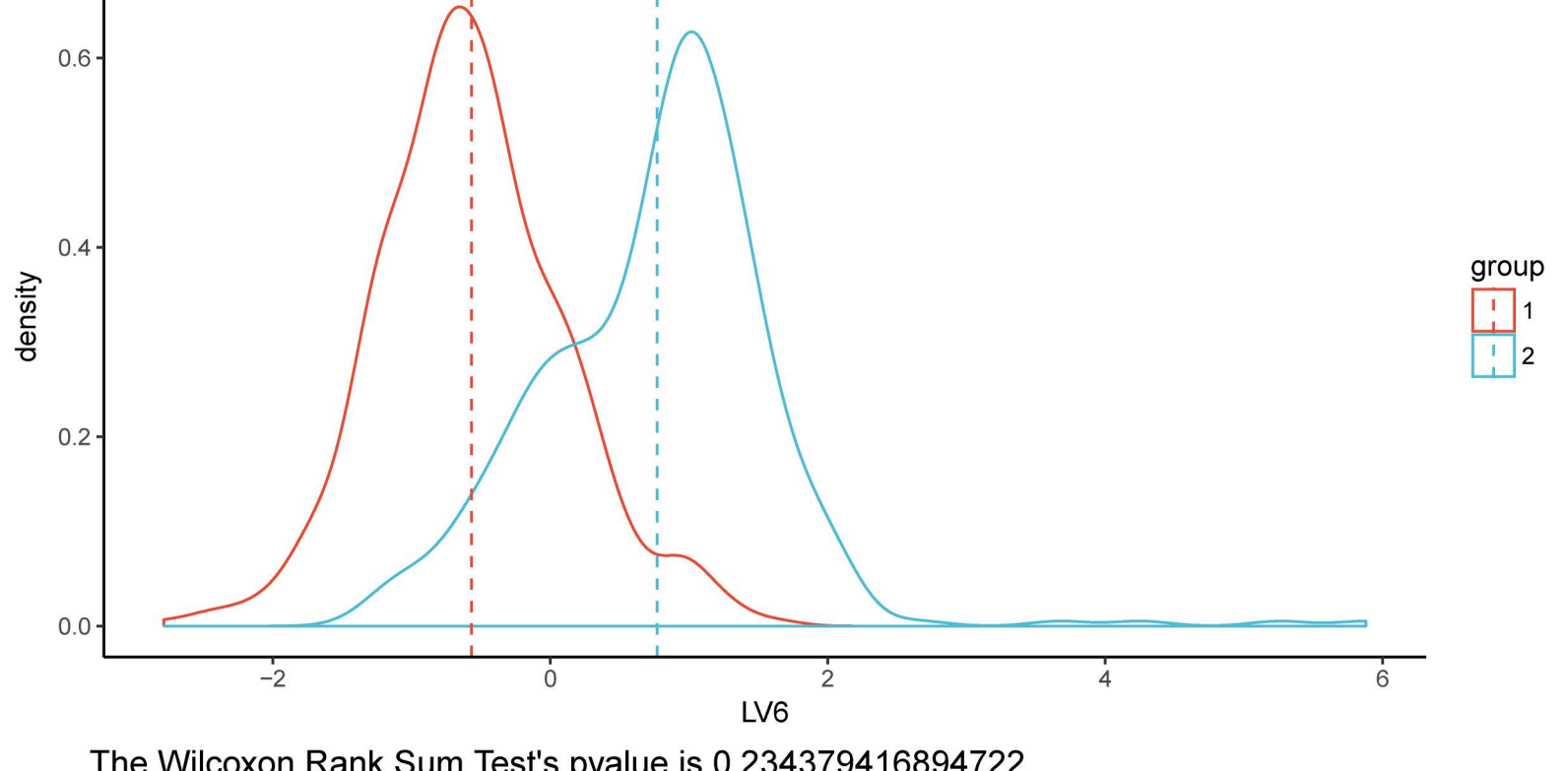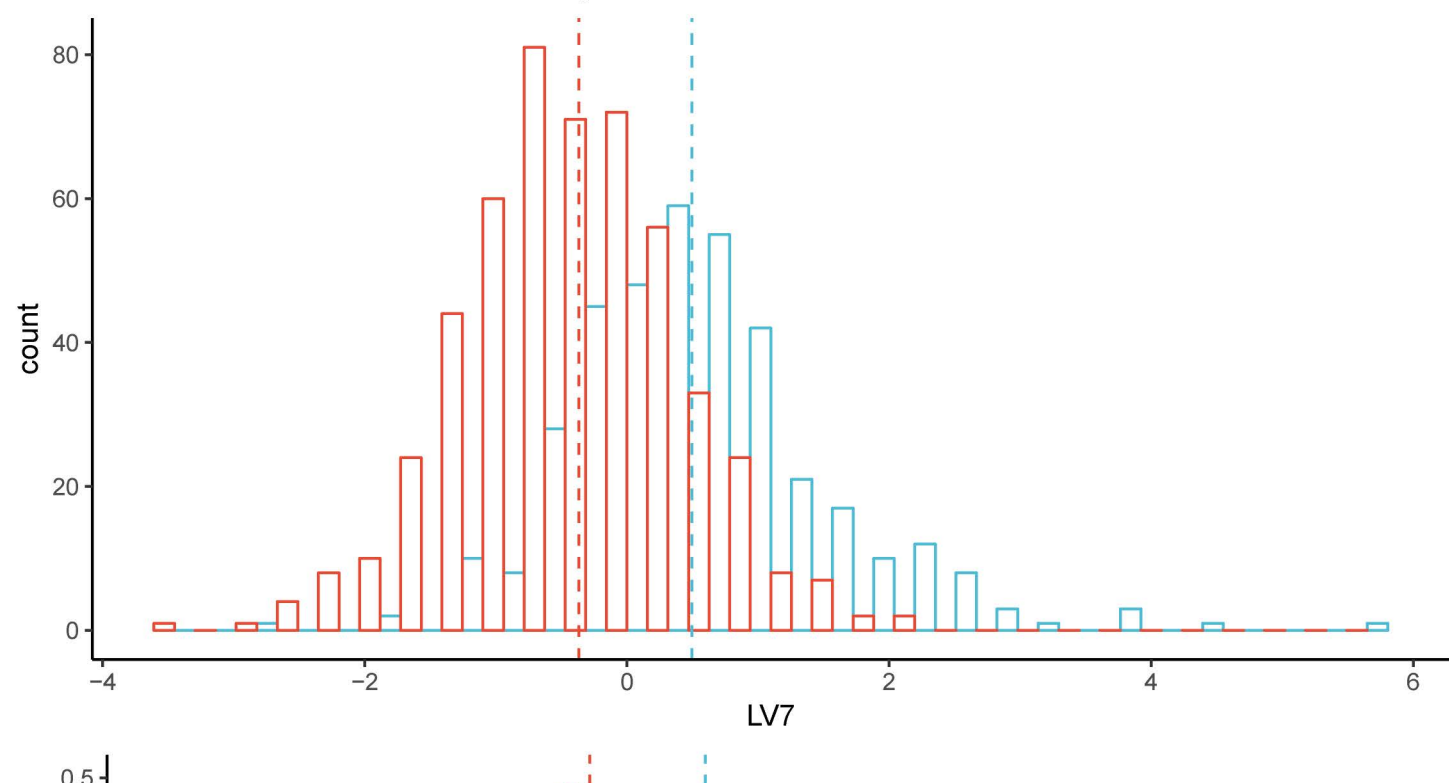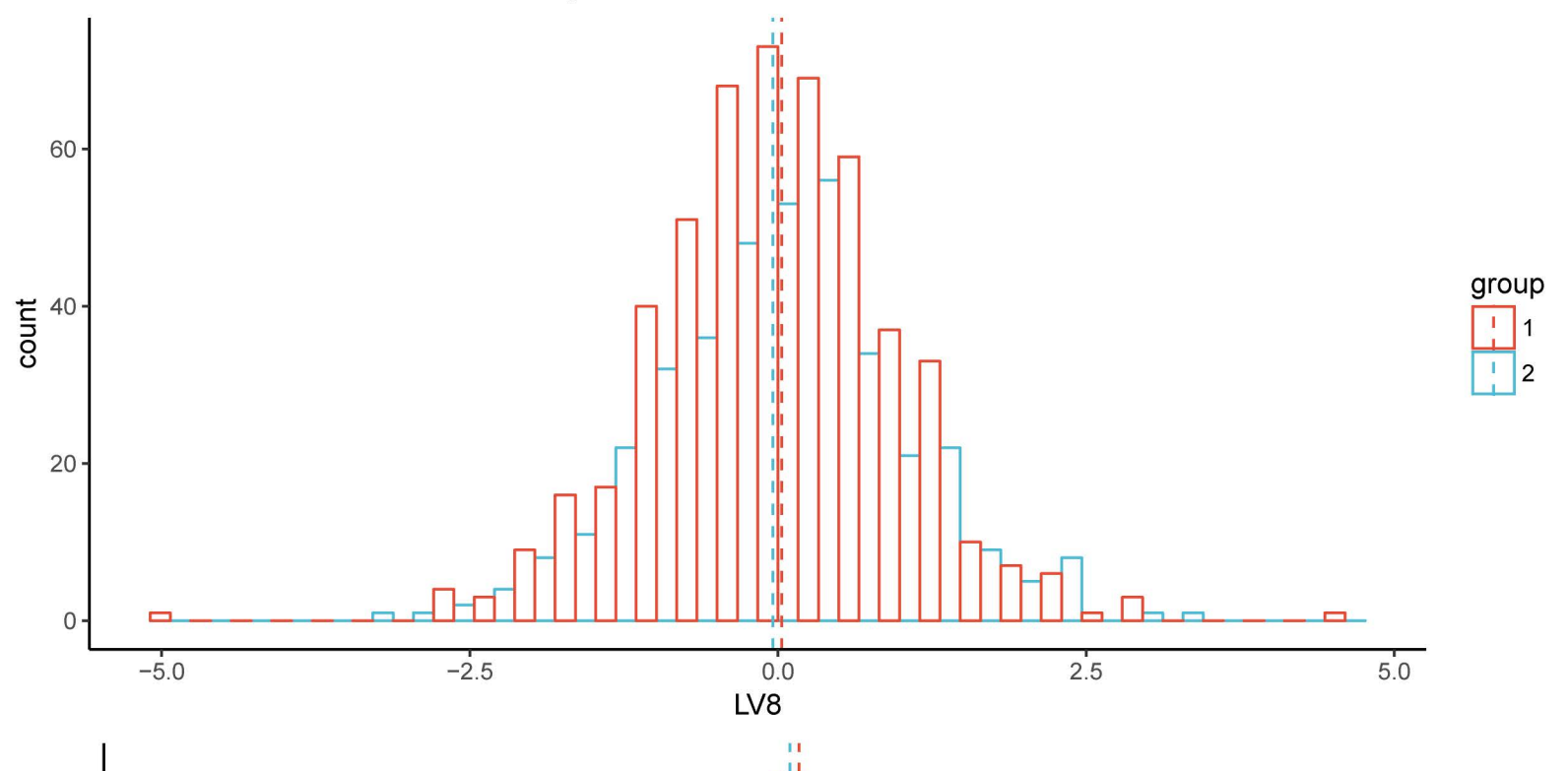

Supplement: Supplementary file 4 — Supplementary Fig. 4 The silhouette plot of k-means clustering on individuals. Silhouette values range from 1 to -1, when silhouette value is close to 1 indicating that the individuals are well clustered. The silhouette plot for k-means clustering showed that observations are well clustered [file 43657_2020_5_MOESM4_ESM.pdf]

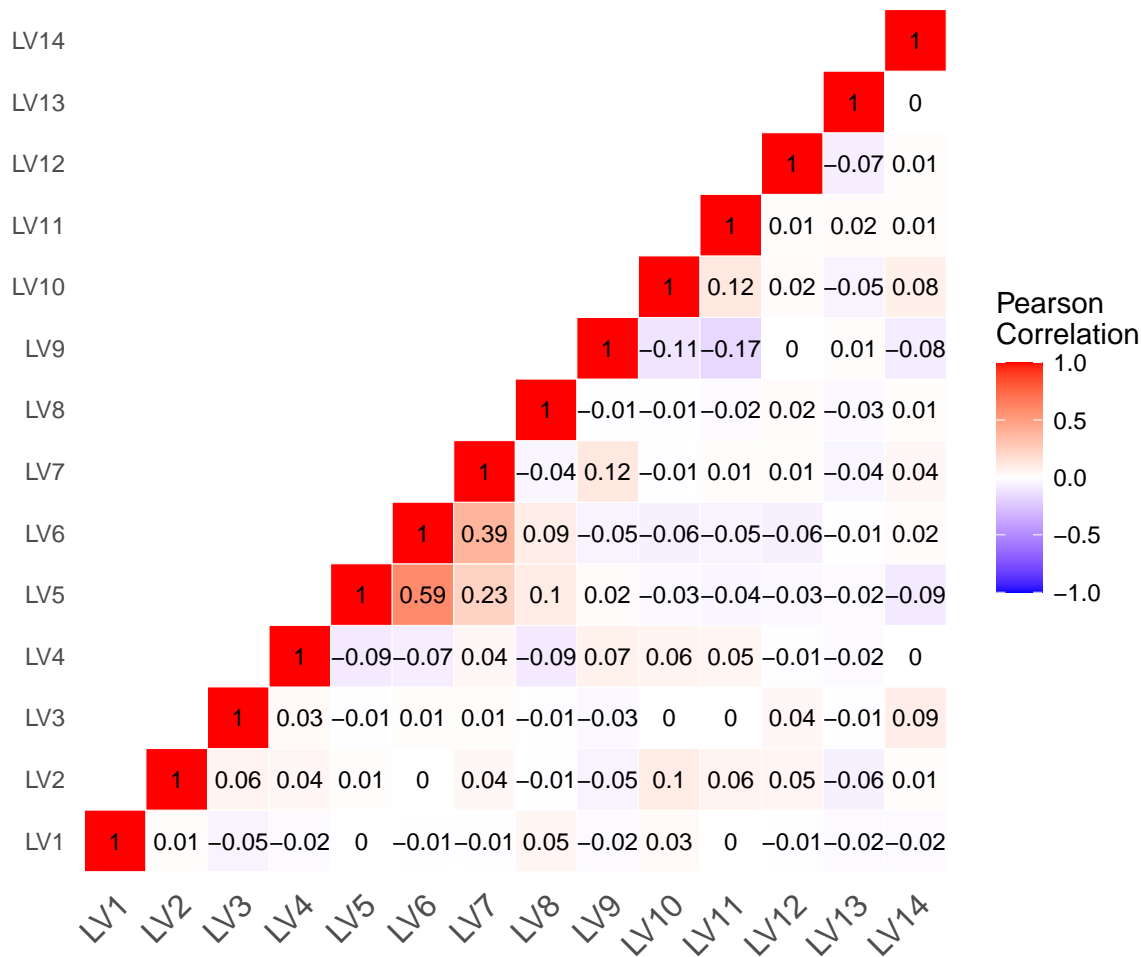

Supplement: Supplementary file 5 — Supplementary Fig. 5 The histogram plot and density plot of each LV (14 LVs) between 2 groups (group 1 with red color and group 2 with blue color). And we further calculated the Wilcoxon Rank Sum test p value (the title of histogram plot) of each LV between 2 groups. [file 43657_2020_5_MOESM5_ESM.pdf]

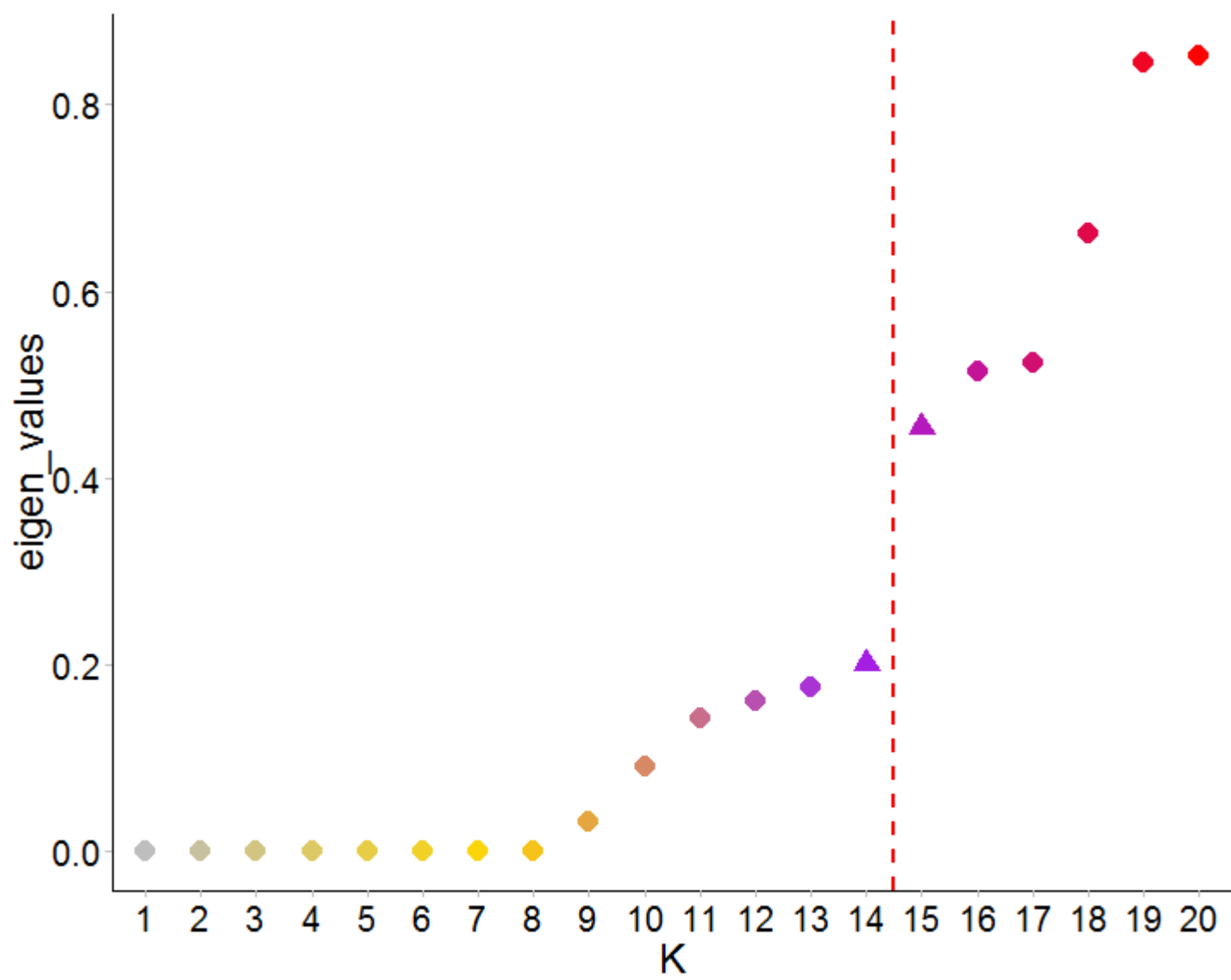

Supplement: Supplementary file 11 — Supplementary file11 (PDF 19 KB) [file 43657_2020_5_MOESM11_ESM.pdf]
