# Supplementary material for: Using Composite Phenotypes to Reveal Hidden Physiological Heterogeneity in High-Altitude Acclimatization in a Chinese Han Longitudinal Cohort
Source: Phenomics. 2021 Feb 22;1(1):3–14. doi: 10.1007/s43657-020-00005-8 (PMC9584130; doi:10.1007/s43657-020-00005-8)
Supplement: Supplementary file 6 — Supplementary Fig. 6 The pairwise Pearson correlation heatmap of 14 composite phenotypes (LV1, … , LV14). The Pearson correlation coefficient ranges from -1 (blue) to 1(red) [file 43657_2020_5_MOESM6_ESM.docx]

| **Supplementary Table1. The loading matrix of principal component analysis of 14 composite phenotypes.** | | | | | | | | | | | | | | |
| --- | --- | --- | --- | --- | --- | --- | --- | --- | --- | --- | --- | --- | --- | --- |
|  | PC1 | PC2 | PC3 | PC4 | PC5 | PC6 | PC7 | PC8 | PC9 | PC10 | PC11 | PC12 | PC13 | PC14 |
| LV1 | -8.53E-04 | 0.02445 | -0.25346 | 0.22933 | -0.3818 | 0.53119 | -0.4201 | 0.39085 | -0.2516 | -0.2426 | 0.02567 | 0.01056 | 0.04927 | 0.00403 |
| LV2 | 0.001283078 | 0.38842 | 0.170716 | 0.26595 | -0.0703 | -0.1727 | -0.5163 | -0.1392 | -0.0403 | 0.53967 | -0.3402 | 0.12346 | 0.06745 | 0.012745 |
| LV3 | -0.009272267 | 0.20814 | 0.179008 | -0.0038 | 0.59227 | -0.0662 | -0.5025 | -0.0498 | -0.0409 | -0.4069 | 0.27204 | -0.2664 | -0.047 | 6.93E-04 |
| LV4 | -0.109106612 | 0.09681 | 0.574501 | -0.0577 | -0.2281 | 0.16385 | -0.0479 | -0.1121 | 0.34772 | -0.3471 | 0.05256 | 0.55279 | 0.04208 | -0.00759 |
| LV5 | 0.587769491 | 0.04862 | -0.09236 | -0.0106 | -0.0718 | -0.1281 | -0.0391 | -0.0615 | 0.02474 | -0.0249 | 0.22826 | 0.09948 | 0.45081 | -0.59176 |
| LV6 | 0.634142017 | 0.10715 | -0.02626 | -0.1021 | 0.02207 | 0.00698 | 0.0199 | 0.0036 | -0.0374 | -0.0309 | 0.04057 | 0.13679 | 0.08037 | 0.738343 |
| LV7 | 0.442135832 | 0.11123 | 0.367529 | -0.0253 | -0.0565 | 0.14132 | 0.11213 | 0.23035 | -0.0123 | 0.02495 | -0.2111 | -0.2379 | -0.6286 | -0.26558 |
| LV8 | 0.121877518 | 0.00927 | -0.46688 | 0.29601 | 0.14387 | 0.13882 | -0.0769 | -0.1608 | 0.72257 | -0.0903 | -0.1685 | 0.03844 | -0.2166 | -0.02589 |
| LV9 | 0.063101913 | -0.4507 | 0.412727 | 0.25526 | -0.0238 | 0.18734 | -0.0514 | 0.07184 | 0.3001 | 0.1644 | -0.0155 | -0.478 | 0.38874 | 0.13035 |
| LV10 | -0.101843304 | 0.48275 | 9.27E-04 | 0.03123 | -0.2175 | 0.23337 | 0.16678 | -0.136 | 0.16547 | 0.29471 | 0.64951 | -0.2485 | -0.0766 | 0.061655 |
| LV11 | -0.096719801 | 0.44245 | -0.05004 | -0.2516 | -0.2983 | -0.2785 | 0.07727 | 0.20337 | 0.23104 | -0.3248 | -0.3463 | -0.3998 | 0.27167 | 0.052396 |
| LV12 | -0.052663335 | 0.13998 | 0.066753 | 0.53014 | 0.1819 | -0.3292 | 0.24808 | 0.63737 | 0.07001 | -0.0011 | 0.17498 | 0.21094 | 0.03624 | 0.039332 |
| LV13 | -0.039115583 | -0.167 | -0.06853 | -0.5793 | 0.03843 | -0.069 | -0.3389 | 0.48685 | 0.33467 | 0.32965 | 0.17771 | 0.12262 | -0.069 | -0.01264 |
| LV14 | -0.045255014 | 0.30021 | 0.015588 | -0.1686 | 0.49544 | 0.56639 | 0.26263 | 0.1477 | -0.022 | 0.15957 | -0.2755 | 0.10886 | 0.31683 | -0.08937 |
