# Supplementary material for: Using Composite Phenotypes to Reveal Hidden Physiological Heterogeneity in High-Altitude Acclimatization in a Chinese Han Longitudinal Cohort
Source: Phenomics. 2021 Feb 22;1(1):3–14. doi: 10.1007/s43657-020-00005-8 (PMC9584130; doi:10.1007/s43657-020-00005-8)
Supplement: Supplementary file 7 — Supplementary file7 (DOCX 15 KB) [file 43657_2020_5_MOESM7_ESM.docx]

| **Supplementary Table2. The Pvalues of pairwise Pearson correlation of the 14 composite phenotypes in Group1** | | | | | | | | | | | | | | |
| --- | --- | --- | --- | --- | --- | --- | --- | --- | --- | --- | --- | --- | --- | --- |
|  | LV1 | LV2 | LV3 | LV4 | LV5 | LV6 | LV7 | LV8 | LV9 | LV10 | LV11 | LV12 | LV13 | LV14 |
| LV1 | 0 | 0.74596 | 0.28209 | 0.70094 | 0.893288 | 0.273017 | 0.27587 | 0.388457 | 0.392237 | 0.85918 | 0.80762 | 0.37363 | 0.81705 | 0.81227 |
| LV2 | 0.74596 | 0 | 0.1319 | 0.18416 | 0.077967 | 0.018957 | 0.443509 | 0.611477 | 0.003406 | 0.00637 | 0.27584 | 0.42644 | 0.07089 | 0.36548 |
| LV3 | 0.28209 | 0.1319 | 0 | 0.62121 | 0.263099 | 0.882698 | 0.500225 | 0.811493 | 0.947893 | 0.70554 | 0.62385 | 0.03957 | 0.12508 | 0.0452 |
| LV4 | 0.70094 | 0.18416 | 0.62121 | 0 | 0.188527 | 0.696412 | 0.093721 | 0.046585 | 0.065297 | 0.16453 | 0.57432 | 0.06604 | 0.65731 | 0.83877 |
| LV5 | 0.89329 | 0.07797 | 0.2631 | 0.18853 | 0 | 4.49E-05 | 8.11E-06 | 0.036336 | 0.203931 | 0.02743 | 0.00669 | 0.703 | 0.55185 | 0.29194 |
| LV6 | 0.27302 | 0.01896 | 0.8827 | 0.69641 | 4.49E-05 | 0 | 0.006135 | 5.10E-07 | 2.41E-05 | 0.20152 | 0.08216 | 0.81028 | 0.00621 | 0.00199 |
| LV7 | 0.27587 | 0.44351 | 0.50023 | 0.09372 | 8.11E-06 | 0.006135 | 0 | 0.194029 | 0.404329 | 0.09186 | 0.00263 | 0.37853 | 0.70712 | 0.00214 |
| LV8 | 0.38846 | 0.61148 | 0.81149 | 0.04659 | 0.036336 | 5.10E-07 | 0.194029 | 0 | 0.913256 | 0.48683 | 0.17041 | 0.15073 | 0.44763 | 0.67935 |
| LV9 | 0.39224 | 0.00341 | 0.94789 | 0.0653 | 0.203931 | 2.41E-05 | 0.404329 | 0.913256 | 0 | 0.05346 | 0.12752 | 0.68991 | 0.80083 | 0.33313 |
| LV10 | 0.85918 | 0.00637 | 0.70554 | 0.16453 | 0.027425 | 0.201524 | 0.091859 | 0.486832 | 0.053459 | 0 | 0.01936 | 0.89772 | 0.17007 | 0.04176 |
| LV11 | 0.80762 | 0.27584 | 0.62385 | 0.57432 | 0.006692 | 0.082163 | 0.002632 | 0.170409 | 0.12752 | 0.01936 | 0 | 0.4281 | 0.26656 | 0.05187 |
| LV12 | 0.37363 | 0.42644 | 0.03957 | 0.06604 | 0.703004 | 0.810283 | 0.378531 | 0.150726 | 0.689907 | 0.89772 | 0.4281 | 0 | 0.13966 | 0.89094 |
| LV13 | 0.81705 | 0.07089 | 0.12508 | 0.65731 | 0.551854 | 0.006214 | 0.707116 | 0.447628 | 0.800831 | 0.17007 | 0.26656 | 0.13966 | 0 | 0.45185 |
| LV14 | 0.81227 | 0.36548 | 0.0452 | 0.83877 | 0.291936 | 0.00199 | 0.002136 | 0.679353 | 0.333129 | 0.04176 | 0.05187 | 0.89094 | 0.45185 | 0 |
