# Supplementary material for: Using Composite Phenotypes to Reveal Hidden Physiological Heterogeneity in High-Altitude Acclimatization in a Chinese Han Longitudinal Cohort
Source: Phenomics. 2021 Feb 22;1(1):3–14. doi: 10.1007/s43657-020-00005-8 (PMC9584130; doi:10.1007/s43657-020-00005-8)
Supplement: Supplementary file 8 — Supplementary file8 (DOCX 15 KB) [file 43657_2020_5_MOESM8_ESM.docx]

| **Supplementary Table3. The Pvalues of pairwise Pearson correlation of the 14 composite phenotypes in Group2** | | | | | | | | | | | | | | |
| --- | --- | --- | --- | --- | --- | --- | --- | --- | --- | --- | --- | --- | --- | --- |
|  | LV1 | LV2 | LV3 | LV4 | LV5 | LV6 | LV7 | LV8 | LV9 | LV10 | LV11 | LV12 | LV13 | LV14 |
| LV1 | 0 | 0.98788 | 0.29509 | 0.5806 | 0.790921 | 0.401554 | 0.332444 | 0.299644 | 0.82289 | 0.17476 | 0.813364 | 0.43717 | 0.16344 | 0.401313 |
| LV2 | 0.98788 | 0 | 0.27622 | 0.90738 | 0.265246 | 0.179454 | 0.002976 | 0.915269 | 0.184529 | 0.57956 | 0.538941 | 0.22756 | 0.3895 | 0.202084 |
| LV3 | 0.29509 | 0.27622 | 0 | 0.12122 | 0.77405 | 0.368862 | 0.808749 | 0.629638 | 0.121425 | 0.39703 | 0.299215 | 0.51585 | 0.08435 | 0.037842 |
| LV4 | 0.5806 | 0.90738 | 0.12122 | 0 | 0.306605 | 0.459843 | 0.07894 | 0.034635 | 0.075772 | 0.50261 | 0.317515 | 0.32733 | 0.62097 | 0.780916 |
| LV5 | 0.79092 | 0.26525 | 0.77405 | 0.30661 | 0 | 5.98E-28 | 9.28E-04 | 5.63E-04 | 4.69E-04 | 0.62993 | 0.177374 | 0.464 | 0.71045 | 0.960311 |
| LV6 | 0.40155 | 0.17945 | 0.36886 | 0.45984 | 5.98E-28 | 0 | 5.35E-05 | 0.165141 | 6.26E-12 | 0.37122 | 3.37E-04 | 0.644 | 0.50886 | 3.33E-04 |
| LV7 | 0.33244 | 0.00298 | 0.80875 | 0.07894 | 9.28E-04 | 5.35E-05 | 0 | 0.833955 | 0.216011 | 0.57023 | 0.032974 | 0.21973 | 0.7711 | 0.149582 |
| LV8 | 0.29964 | 0.91527 | 0.62964 | 0.03463 | 5.63E-04 | 0.165141 | 0.833955 | 0 | 0.993694 | 0.58403 | 0.708251 | 0.3324 | 0.4269 | 0.317771 |
| LV9 | 0.82289 | 0.18453 | 0.12142 | 0.07577 | 4.69E-04 | 6.26E-12 | 0.216011 | 0.993694 | 0 | 0.18591 | 6.51E-04 | 0.1456 | 0.19829 | 0.217305 |
| LV10 | 0.17476 | 0.57956 | 0.39703 | 0.50261 | 0.629925 | 0.371218 | 0.570235 | 0.584028 | 0.185915 | 0 | 0.207767 | 0.45477 | 0.22681 | 0.925006 |
| LV11 | 0.81336 | 0.53894 | 0.29922 | 0.31752 | 0.177374 | 3.37E-04 | 0.032974 | 0.708251 | 6.51E-04 | 0.20777 | 0 | 0.49401 | 0.16524 | 0.132228 |
| LV12 | 0.43717 | 0.22756 | 0.51585 | 0.32733 | 0.464001 | 0.644001 | 0.219734 | 0.332397 | 0.145602 | 0.45477 | 0.494015 | 0 | 0.04515 | 0.630949 |
| LV13 | 0.16344 | 0.3895 | 0.08435 | 0.62097 | 0.710454 | 0.508857 | 0.771104 | 0.426896 | 0.198287 | 0.22681 | 0.165239 | 0.04515 | 0 | 0.445511 |
| LV14 | 0.40131 | 0.20208 | 0.03784 | 0.78092 | 0.960311 | 3.33E-04 | 0.149582 | 0.317771 | 0.217305 | 0.92501 | 0.132228 | 0.63095 | 0.44551 | 0 |
