# Supplementary material for: Using Composite Phenotypes to Reveal Hidden Physiological Heterogeneity in High-Altitude Acclimatization in a Chinese Han Longitudinal Cohort
Source: Phenomics. 2021 Feb 22;1(1):3–14. doi: 10.1007/s43657-020-00005-8 (PMC9584130; doi:10.1007/s43657-020-00005-8)
Supplement: Supplementary file 9 — Supplementary file9 (DOCX 16 KB) [file 43657_2020_5_MOESM9_ESM.docx]

| **Supplementary Table 4. The Pvalues of the difference of Pearson correlations between the two clusters using fisher's z transformation tests.** | | | | | | | | | | | | | | |
| --- | --- | --- | --- | --- | --- | --- | --- | --- | --- | --- | --- | --- | --- | --- |
|  | **LV1** | **LV2** | **LV3** | **LV4** | **LV5** | **LV6** | **LV7** | **LV8** | **LV9** | **LV10** | **LV11** | **LV12** | **LV13** | **LV14** |
| **LV1** | NaN | 8.24E-01 | 9.25E-01 | 8.66E-01 | 9.09E-01 | 1.77E-01 | 1.49E-01 | 8.22E-01 | 4.67E-01 | 3.61E-01 | 7.36E-01 | 2.43E-01 | 2.27E-01 | 6.30E-01 |
| **LV2** | 8.24E-01 | NaN | 8.77E-01 | 4.37E-01 | 7.62E-01 | 6.10E-01 | 7.88E-02 | 6.81E-01 | 3.55E-03 | 1.75E-01 | 8.08E-01 | 6.91E-01 | 6.00E-01 | 1.20E-01 |
| **LV3** | 9.25E-01 | 8.77E-01 | NaN | 1.34E-01 | 6.09E-01 | 4.37E-01 | 5.34E-01 | 6.02E-01 | 2.57E-01 | 3.74E-01 | 2.68E-01 | 6.67E-02 | 2.10E-02 | 7.86E-01 |
| **LV4** | 8.66E-01 | 4.37E-01 | 1.34E-01 | NaN | 9.36E-01 | 7.59E-01 | 8.09E-01 | 7.59E-01 | 8.83E-01 | 6.92E-01 | 6.95E-01 | 5.24E-02 | 9.31E-01 | 9.37E-01 |
| **LV5** | 9.09E-01 | 7.62E-01 | 6.09E-01 | 9.36E-01 | NaN | 4.31E-09 | 5.58E-08 | 2.08E-01 | 6.68E-02 | 2.84E-01 | 4.57E-01 | 7.59E-01 | 9.16E-01 | 4.69E-01 |
| **LV6** | 1.77E-01 | 6.10E-01 | 4.37E-01 | 7.59E-01 | 4.31E-09 | NaN | 1.98E-01 | 2.57E-02 | 1.21E-02 | 8.78E-01 | 1.11E-01 | 6.12E-01 | 2.23E-02 | 4.76E-01 |
| **LV7** | 1.49E-01 | 7.88E-02 | 5.34E-01 | 8.09E-01 | 5.58E-08 | 1.98E-01 | NaN | 3.15E-01 | 1.38E-01 | 5.05E-01 | 7.32E-01 | 7.21E-01 | 6.42E-01 | 3.63E-01 |
| **LV8** | 8.22E-01 | 6.81E-01 | 6.02E-01 | 7.59E-01 | 2.08E-01 | 2.57E-02 | 3.15E-01 | NaN | 9.39E-01 | 3.85E-01 | 2.39E-01 | 9.47E-02 | 9.14E-01 | 3.04E-01 |
| **LV9** | 4.67E-01 | 3.55E-03 | 2.57E-01 | 8.83E-01 | 6.68E-02 | 1.21E-02 | 1.38E-01 | 9.39E-01 | NaN | 7.99E-01 | 1.10E-01 | 1.73E-01 | 2.54E-01 | 7.60E-01 |
| **LV10** | 3.61E-01 | 1.75E-01 | 3.74E-01 | 6.92E-01 | 2.84E-01 | 8.78E-01 | 5.05E-01 | 3.85E-01 | 7.99E-01 | NaN | 5.70E-01 | 5.15E-01 | 9.81E-01 | 2.10E-01 |
| **LV11** | 7.36E-01 | 8.08E-01 | 2.68E-01 | 6.95E-01 | 4.57E-01 | 1.11E-01 | 7.32E-01 | 2.39E-01 | 1.10E-01 | 5.70E-01 | NaN | 3.01E-01 | 7.57E-02 | 1.60E-02 |
| **LV12** | 2.43E-01 | 6.91E-01 | 6.67E-02 | 5.24E-02 | 7.59E-01 | 6.12E-01 | 7.21E-01 | 9.47E-02 | 1.73E-01 | 5.15E-01 | 3.01E-01 | NaN | 5.76E-01 | 6.50E-01 |
| **LV13** | 2.27E-01 | 6.00E-01 | 2.10E-02 | 9.31E-01 | 9.16E-01 | 2.23E-02 | 6.42E-01 | 9.14E-01 | 2.54E-01 | 9.81E-01 | 7.57E-02 | 5.76E-01 | NaN | 2.85E-01 |
| **LV14** | 6.30E-01 | 1.20E-01 | 7.86E-01 | 9.37E-01 | 4.69E-01 | 4.76E-01 | 3.63E-01 | 3.04E-01 | 7.60E-01 | 2.10E-01 | 1.60E-02 | 6.50E-01 | 2.85E-01 | NaN |
