# Supplementary material for: Using Composite Phenotypes to Reveal Hidden Physiological Heterogeneity in High-Altitude Acclimatization in a Chinese Han Longitudinal Cohort
Source: Phenomics. 2021 Feb 22;1(1):3–14. doi: 10.1007/s43657-020-00005-8 (PMC9584130; doi:10.1007/s43657-020-00005-8)
Supplement: Supplementary file 10 — Supplementary file10 (DOCX 15 KB) [file 43657_2020_5_MOESM10_ESM.docx]

| **Supplementary Table 5. R output of Linear Regression Model 2 (based on LVs).** |
| --- |
|  |
| Call: |
| lm(formula = SPO2 ~ ., data = dt_LV) |
|  |
| Residuals: |
| Min 1Q Median 3Q Max |
| -15.706 -2.408 -0.075 2.173 36.161 |
|  |
| Coefficients: |
| Estimate Std. Error t value Pr(>\|t\|) |
| (Intercept) -11.94677 0.14239 -83.899 <2e-16 *** |
| LV1 -0.08154 0.14288 -0.571 0.5683 |
| LV2 -0.20616 0.14402 -1.431 0.1527 |
| LV3 -0.01616 0.14360 -0.113 0.9104 |
| LV4 -0.07377 0.14476 -0.510 0.6105 |
| LV5 -0.07387 0.17832 -0.414 0.6788 |
| LV6 0.02890 0.18976 0.152 0.8790 |
| LV7 -0.15047 0.15815 -0.951 0.3417 |
| LV8 -0.14114 0.14431 -0.978 0.3283 |
| LV9 0.04234 0.14821 0.286 0.7752 |
| LV10 -0.18750 0.14566 -1.287 0.1984 |
| LV11 0.12644 0.14601 0.866 0.3867 |
| LV12 -0.28773 0.14309 -2.011 0.0446 * |
| LV14 0.04097 0.14531 0.282 0.7781 |
| --- |
| Signif. codes: 0 ‘***’ 0.001 ‘**’ 0.01 ‘*’ 0.05 ‘.’ 0.1 ‘ ’ 1 |
|  |
| Residual standard error: 4.231 on 869 degrees of freedom |
| Multiple R-squared: 0.01395, Adjusted R-squared: -0.0007972 |
| F-statistic: 0.946 on 13 and 869 DF, p-value: 0.5042 |
|  |
